# The role of *Pygo2* for Wnt/ß-catenin signaling activity during intestinal tumor initiation and progression

**DOI:** 10.18632/oncotarget.13016

**Published:** 2016-11-02

**Authors:** Suranand B. Talla, Felix H. Brembeck

**Affiliations:** ^1^ Tumor Biology and Signal Transduction, Dept. of Hematology and Medical Oncology, Georg-August-University Göttingen, Germany

**Keywords:** colon cancer, Wnt/ß-catenin signaling, adenomatous polyposis coli, ß-catenin, Pygo2

## Abstract

*Pygo2* acts as a co-activator of Wnt signaling in a nuclear complex with ß-catenin/BCL9/BCL9-2 to increase target gene transcription. Previous studies showed that *Pygo2* is upregulated in murine intestinal tumors and human colon cancer, but is apparently dispensable for normal intestinal homeostasis. Here, we have evaluated the *in vivo* role of *Pygo2* during intestinal tumorigenesis using *Pygo2* deficient mice. We analyzed chemically induced colon tumor development and conditional intestine specific mouse models harboring either *Apc* loss-of-function (LOF) or *Ctnnb1* gain-of-function (ß-catenin GOF). Remarkably, the number and size of chemically induced tumors was significantly reduced in *Pygo2* deficient mice, suggesting that *Pygo2* has a tumor promoting function. Furthermore, loss of *Pygo2* rescued early tumorigenesis of *Ctnnb1* GOF mutants. In contrast, *Pygo2* ablation was not sufficient to prevent tumor development of *Apc* LOF mice. The effect on tumor formation by *Pygo2* knockout was linked to the repression of specific deregulated Wnt target genes, in particular of *c-Myc*. Moreover, the role of *Pygo2* appears to be associated with the signaling output of deregulated Wnt signaling in the different tumor models. Thus, targeting *Pygo2* might provide a novel strategy to suppress tumor formation in a context dependent manner.

## INTRODUCTION

The members of the PYGOPUS and BCL9 protein families have been discovered as novel co-factors of canonical Wnt/ß-catenin signaling [1–3]. These co-activators form a complex with ß-catenin-LEF/TCF to activate transcription of Wnt target genes and to enhance the signaling output [4–8]. However, the knockout of *Pygopus* and *Bcl9's* in the mouse has resulted in only limited embryonic defects compared to other components of the Wnt/ß-catenin pathway [9, 10]. Therefore, PYGOPUS and BCL9 proteins may not be absolutely essential for Wnt/ß-catenin signaling during embryonic development in vertebrates. On the other hand, several studies revealed a specific role in particular for *Pygo2* and *Bcl9-2* in diseases such as colon cancer to hyperactivate canonical Wnt signaling [3, 8]. Deregulation of the Wnt/ß-catenin signaling pathway was shown to be the primary driver of colon cancer development: the vast majority of sporadic human colon cancers harbor loss-of-function (*LOF*) mutations of the adenomatous polyposis coli gene *(Apc)* and less frequently gain of function (*GOF*) mutations of the ß-catenin encoding proto-oncogene *Ctnnb1* [11]. These mutations constitutively activate Wnt/ß-catenin signaling by preventing ß-catenin degradation, which results in accumulation and nuclear translocation of stabilized ß-catenin. In the nucleus, ß-catenin interacts with TCF/LEF transcription factors to activate target gene transcription [12, 13].

Overactivated Wnt/ß-catenin signaling in colon cancer may be further increased by deregulated expression of different members of the *Pygopus* and *Bcl9* coactivator family. Indeed, our previous studies demonstrated significant overexpression of PYGO2 in tumors of *APC^Min/+^* mice, colon cancer cells and in human colon cancer, indicating a possible role in tumor development. In addition, *Pygo2* knockdown in colon cancer cells is able to suppress Wnt target gene transcription [8]. Our and other previous *in vitro* and *in vivo* studies suggested that *Bcl9-2* also acts as an oncogene that enhances Wnt signaling activity in cancer [8, 9, 14–16]. Besides colon cancer, *Pygo2* was also suggested to have a potential role in various other malignancies including breast, ovarian, lung, glioblastomas and liver cancers [17–21].

However, the functional relevance of the *Pygopus* homologues during different stages of colon cancer has not yet been studied *in vivo*. From our previous studies, we found that BCL9, BCL9-2 and PYGO2, but not PYGO1, were expressed in the epithelial cells of the normal intestinal mucosa [8]. Despite PYGO2 overexpression in colon cancer, our preliminary studies revealed that the knockout of *Pygo2* in the intestine had no influence on embryonic intestinal development and for adult intestinal homeostasis (Schelp and Brembeck, unpublished data). Therefore, the present study was designed to investigate the role of *Pygo2* during colon cancer initiation and progression *in vivo*.

Mouse models are valuable tools to study the crucial events during initiation and progression of colon cancer. Several mouse models of *Apc LOF* and *Ctnnb1 GOF* were engineered that mimic the different stages of human colon cancer development [22]. Intercrossing of these mouse models with other mutants revealed the importance of several additional genes for colon carcinogenesis [23]. In our study, we first analyzed a chemically induced colon cancer mouse model with and without constitutive knockout of *Pygo2*. In addition, we have generated inducible compound mutant mice to analyze the role of *Pygo2* during different stages of tumorigenesis. For this, we have intercrossed mice harboring either hetero- or homozygous intestine specific deletion of *Pygo2* with inducible mutants of stabilized ß-catenin (*Ctnnb1*) [24] or homo- and heterozygous truncated *Apc* [25]. Our results presented here indicate that *Pygo2* synergizes intestinal tumor formation *in vivo* that is primarily driven by aberrant Wnt signaling. We describe that *Pygo2* knockout reduced tumor formation in chemically induced colon tumors. Moreover, we show that *Pygo2* is essential for early stages of intestinal tumorigenesis induced by *Ctnnb1* mutation, but not in the context of *Apc* mutations. Thus, targeting *Pygo2* may represent an attractive therapeutic option to suppress or arrest tumor growth in human colon cancer in a context dependent manner. This is of particular interest, since *Pygo2* appears to be dispensable for normal intestinal homeostasis.

## RESULTS

### *Pygo2* knockout delays the progression of chemically induced colon tumors

Our previous data indicated that *Pygo2* might be important for intestinal tumorigenesis [1, 8, 26]. We have previously analyzed in detail the phenotype of mutant mice with constitutive deletion of both *Pygo2* alleles in the intestine (Schelp and Brembeck, unpublished data) and compared them with control littermates (see Methods, Schäffer and Birchmeier). Analysis of mutant mice revealed that loss of *Pygo2* neither disturbed normal embryonic development of the intestine nor impaired intestinal homeostasis or lineage commitment in adults (Schelp and Brembeck, unpublished data). Of note, we also did not observe any effect of intestinal deletion of both *Pygo1* and *Pygo2* genes, indicating that both genes are completely dispensable for normal intestinal function (Schelp and Brembeck, unpublished data).

To test the potential pro-oncogenic role of *Pygo2* in intestinal tumors, we first challenged constitutive, intestine specific *Pygo2* deficient mice (“*Pygo2^−/−^”;* corresponding to the genotype *Vil^Cre^; Pygo2 ^Δ/^*^*Δ*^) and control littermates (*Pygo2^lox(ex3)/lox(ex3)^*). We treated the mice with an adapted scheme for chemically induced tumor formation in the colon [27]. 6- to 8-week old mice were injected intraperitoneally with one single dose of azoxymethane (AOM). One week later, mice were treated with dextran sodium sulfate (DSS) in the drinking water for five consecutive days. Additionally, control and *Pygo2* knockout mice were treated with DSS alone, to analyze a role of *Pygo2* during intestinal epithelial regeneration following inflammation. However, independent of *Pygo2* loss, all mice recovered from the initial phase of DSS induced inflammation as monitored by changes in body weight and survival (Supplementary Figure S1A and data not shown). Our histopathological analysis at 14 and 28 days after beginning the treatment did not reveal any disturbance of intestinal regeneration by *Pygo2* ablation (Supplementary Figure S1B, S1C). These observations corroborate our results that *Pygo2* is apparently dispensable for normal intestinal homeostasis in the adult.

**Figure 1:**
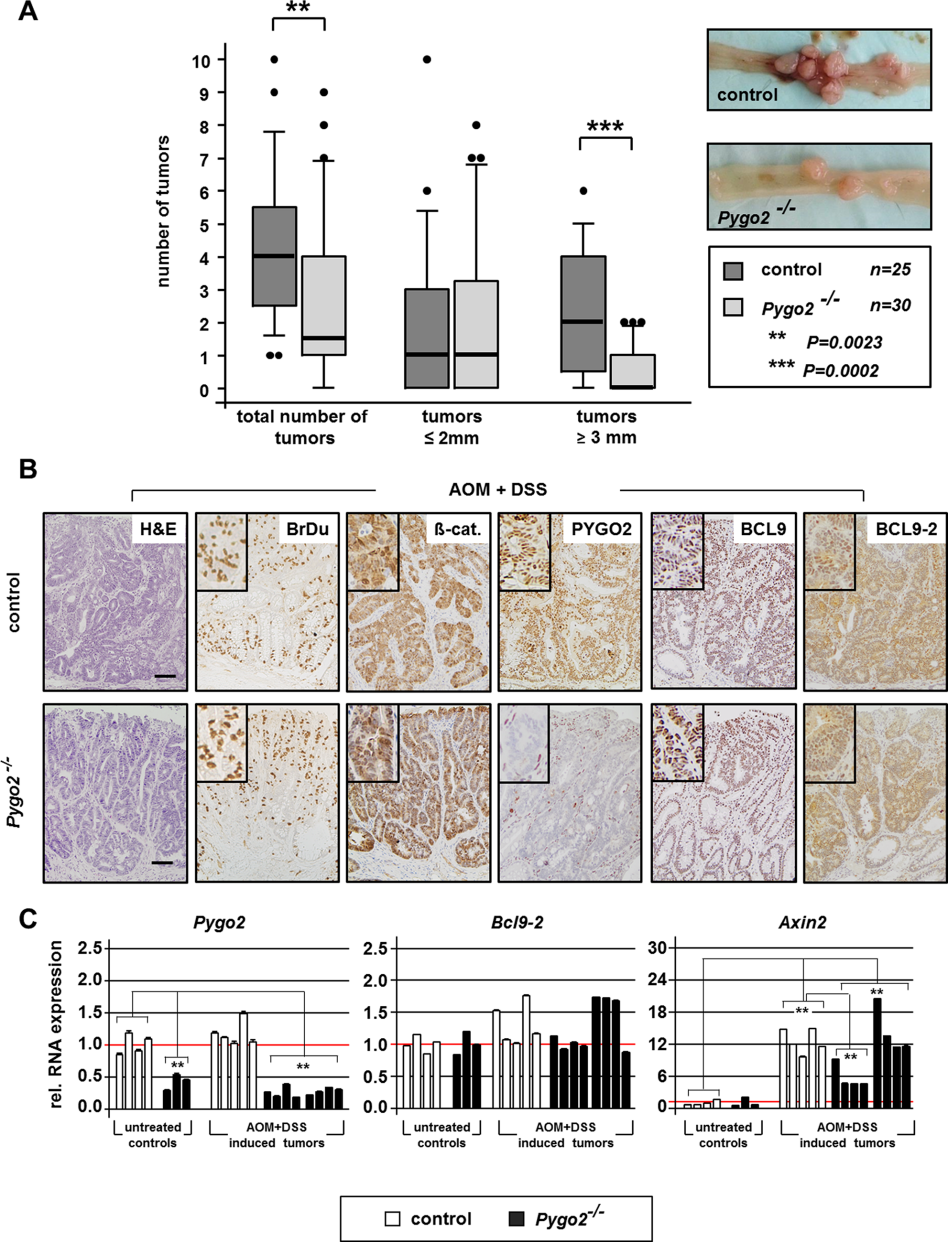
Knockout of *Pygo2* delays the progression of chemically induced intestinal tumors (**A**) Boxplot analysis illustrates number and size of colon tumors in control (*Pygo2^lox(ex3)/lox(ex3)^*; *n* = 30) and constitutive *Pygo2* deficient mice (marked with Pygo2^−/−^ corresponding to *Vil^Cre^; Pygo2^Δ/Δ^; n* = 35). All mice were sacrificed six months after beginning with AOM and DSS treatment. *Pygo2* deficient animals were compared to age-matched littermate controls. Significant difference is indicated with ** for *P* < 0.01 and *** for *P* < 0.001. Microscopic views of control and *Pygo2* deficient colon tumors show tumor burden. (**B**) Representative H&E stains and immunostains with indicated antibodies on tumor sections from control and *Pygo2* knockout animals. Scale bars in the pictures represent 200 μm for all IHC. Inserts show the staining at higher magnification. (**C**) qRT-PCR analyses of RNA extracted from colon tumors of control and *Pygo2* deficient animals and colon tissues obtained from untreated mice with the same genotype. Each bar represents the relative RNA expression of the indicated gene for single animal; each result represents at least three independent experiments. Significances were calculated for the mean expression level compared to untreated controls and relative to the expression levels of control tumors, respectively. All significant differences are marked with * for *P* < 0.05 and ** for *P* < 0.01.

We monitored survival and tumor development in chemically treated mice for six months. During this time, most animals developed massive signs of anal bleeding and weight loss. In total, 86 percent of control mice (25 of 29 animals) and 83 percent of *Pygo2* knockout animals (30 of 36 mice) were available for analysis at this endpoint. Anal prolapse was seen in 18 of 25 control mice (72%), but only in 5 of 30 *Pygo2* deficient animals (17%). We carefully examined the entire intestine and measured the size of tumors, which were all located within the colon (Figure 1A). The total number of colon tumors was highly significantly reduced in *Pygo2* mutant animals (mean number of tumors 2.5 in *Pygo2* knockouts versus 4.2 in controls, *P* = 0.0023; Figure 1A). Moreover, the number of larger tumors (3 mm diameter or more) was also strongly reduced compared to controls (0.5 versus 2.2, *P* = 0.0002; Figure 1A).

We further analyzed the tumors by immunohistochemistry and performed qRT-PCR using total RNA extracted from tumor tissues and whole tissues derived from the colon of untreated controls (Figure 1B, 1C). Colon tumors from controls and *Pygo2* knockout animals showed partially invasiveness into the submucosa and high proliferation as detected by BrdU stains (Figure 1B). Most tumors also demonstrated nuclear ß-catenin, which suggests that chemically induced tumors are linked to hyperactivated Wnt/ß-catenin signaling. PYGO2 was strongly expressed in the nuclei of the tumor cells in controls, but not detectable in tumors of *Pygo2* deficient mice. On the RNA level, *Pygo2* was not transcriptionally induced in the tumors, and significantly downregulated in *Pygo2* knockout tumors. We also analyzed the expression of the co-factors *Bcl9* and *Bcl9- 2*. The expression of BCL9 was NOT increased as we have previously reported in human colon cancers [8]. BCL9-2 was moderately upregulated in chemically induced colon tumors (Figure 1B, 1C). This is in contrast to our previous observation that BCL9-2 expression is strongly induced in adenoma of *APC^Min/+^* mice and human colon cancer [8].

Moreover, *Axin2*, representing a reliable marker of activated Wnt/ß-catenin signaling [28], was strongly transcriptionally induced in the tumors of control and *Pygo2* deficient animals. Of note, a subgroup of colon tumors derived from *Pygo2* deficient animals showed significant repression of *Axin2*, suggesting that Wnt/ß-catenin signaling is partially inhibited in these tumors of *Pygo2* deficient mice.

Taken together, ablation of *Pygo2* can partially rescue the tumor formation of chemically induced colon tumors that may be linked to hyperactivated Wnt/ß-catenin signaling. Thus, loss of *Pygo2* cannot prevent colon tumor development in the chemically induced carcinogenesis model, but may delay tumor progression in terms of tumor number and size.

### Deletion of *Pygo2* rescues the initial phase of intestinal hyperproliferation induced by ß-catenin *(Ctnnb1)* gain-of-function, but not hyperproliferation and adenoma formation induced by *Apc* loss-of-function

Our preliminary results indicated that deletion of *Pygo2* exerts different effects in early phases of intestinal tumor development in contrast to APC^Min/+^ mice, in which the formation of adenoma was not prevented by constitutive ablation of *Pygo2* (Schelp and Brembeck, unpublished data). In our study reported here, we used inducible intestine-specific ablation of either one or both alleles of *Pygo2* (hereafter labeled with *Pygo2^+/−^* and *Pygo2^−/−^;* corresponding to the genotypes *Vil^Cre-ERT^; Pygo2 ^Δ/+^* and *Vil^Cre-ERT^; Pygo2 ^Δ/Δ^* respectively). Pygo2 deficient mice were crossed with genetic mutants harboring either hetero- or homozygous truncated *Apc (Apc* LOF*)* [25] or heterozygous stabilized ß-catenin *(Ctnnb1 GOF)* to generate compound animals [24]. Details on mutant mice are provided in Methods. We induced genetic recombination in the intestine by intraperitoneal injection of tamoxifen for five consecutive days [29]. With this, we achieved almost complete deletion of *Pygo2* expression throughout the small and large intestine (data not shown) and introduced *Apc* or *Ctnnb1* mutations in the same targeted epithelial cells. In all studies, *Pygo2* deficient animals were compared to appropriate control littermates with the corresponding genetic background.

**Figure 2:**
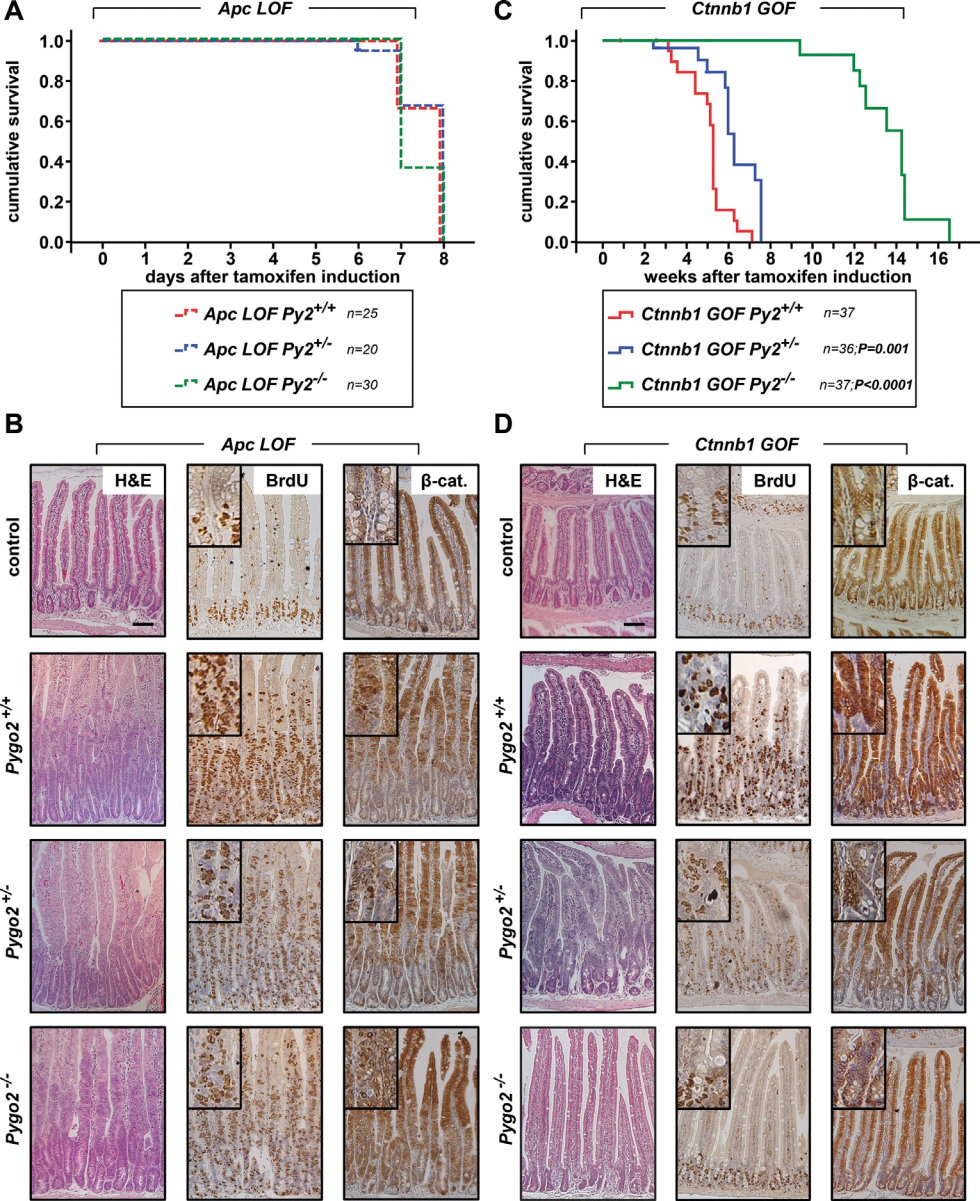
Knockout of *Pygo2* completely rescues the initial phase of intestinal hyperproliferation induced by ß-catenin (*Ctnnb1*) gain-of-function, but not of *Apc* loss-of-function (**A**) Kaplan-Meier survival analysis of compound homozygous APC loss-of-function animals (*LOF*) with wild type *Pygo2* expression (labelled with *Apc* LOF Py2^+/+^ corresponding to the genotype *Vil^Cre-ERT^; Apc^Δex15/Δex15^; Pygo2^+/+^, n* = 25) compared to heterozygous *Pygo2* deficient mice (marked with *Apc* LOF Py2^+/−^ corresponding to *Vil^Cre-ERT^; Apc^Δex15/Δex15^; Pygo2 ^Δ/+^; n* = 20) and homozygous *Pygo2* knockout mice (labelled with *Apc* LOF Py2^−/−^ corresponding to *Vil^Cre-ERT^; Apc^Δex15/Δex15^; Pygo2 ^Δ/Δ^*; *n* = 30). All mice were induced by tamoxifen injection for five consecutive days. (**B**) Representative immunostains on intestinal sections from compound homozygous *Apc LOF* mutant animals with wild type *Pygo2* expression (*Pygo2*^+/+^) compared to mice with hetero- and homozygous ablation of *Pygo2* (*Pygo2*^+/−^ and *Pygo2*^−/−^). The upper panel shows intestinal tissues from uninduced control mice (with the genotype *Apc^lox(ex15)/lox(ex15)^; Pygo2^lox(ex3)/lox(ex3)^).* Animals were analyzed on day six after start of induction with tamoxifen. Tissue sections were stained by H&E and with indicated antibodies. (**C**) Kaplan-Meier survival analysis of compound heterozygous ß-catenin gain-of-function animals (*GOF*) with wild type *Pygo2* expression (labelled with *Ctnnb1* GOF Py2^+/+^ corresponding to the genotype *Vil^Cre-ERT^; Ctnnb1^Δex3/+^; Pygo2^+/+^; n* = 37) compared to heterozygous *Pygo2* deficient mice (marked with *Ctnnb1* GOF Py2^+/−^ corresponding to *Vil^Cre-ERT^; Ctnnb1^Δex3/+^; Pygo2 ^Δ/+^; n* = 36) and homozygous *Pygo2* knockout mice (labelled with *Ctnnb1* GOF Py2^−/−^ corresponding to *Vil^Cre-ERT^; Ctnnb1^Δex3/+^; Pygo2 ^Δ/Δ^*; *n* = 37). Recombination was induced by tamoxifen injections on five consecutive days. Significance was calculated using the P Log Rank Test with *p* = 0.001 for heterozygous and *p* < 0.0001 for homozygous *Pygo2* deficient mice compared to wild type *Pygo2* expressing animals. (**D**) Immunostains with the indicated antibodies on intestinal tissue sections from *Ctnnb1 GOF* animals with different *Pygo2* background. The upper panel shows stainings from uninduced control mice (*Ctnnb1^lox(ex3)/+^; Pygo2^lox(ex3)/lox(ex3)^).* Mice were analyzed on day 18 after beginning of tamoxifen treatment. Scale bars in the pictures represent 200 μm for all IHC. Inserts show the staining at higher magnification.

**Figure 3:**
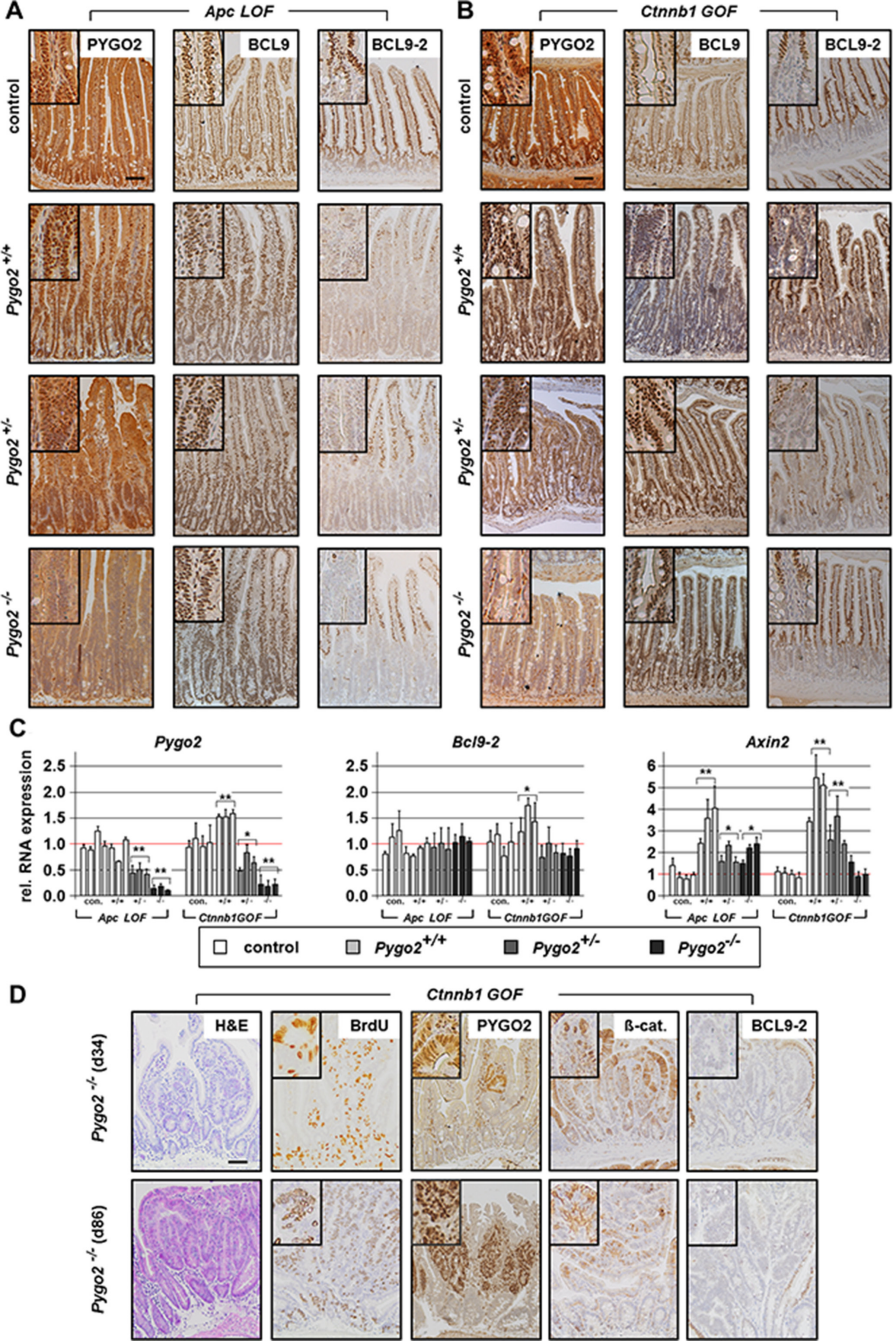
Analysis of *Pygo2*, *Bcl9* co-factors, and *Axin2* in *Apc LOF* and *Ctnnb1 GOF mutants*; failed *Pygo2* deletion by recombination induced microadenoma formation in *Ctnnb1 GOF-Pygo2* deficient animals (**A**, **B**) Immunostainings with indicated antibodies on intestinal tissue sections from compound *Apc* homozygous *LOF* mice (A) and heterozygous *Ctnnb1 GOF* animals (B) with wild type *Pygo2* expression and hetero- and homozygous *Pygo2* ablation. The upper panel shows the same stains from controls. (**C**) qRT-PCR analyses show RNA expression of *Pygo2, Bcl9-2* and *Axin2.* RNA is extracted from intestinal epithelial cells of *Apc LOF* mutants on day 6 and *Ctnnb1 GOF* mutants on day 18 post induction. Each bar represents one animal. Significances were calculated for the mean expression level compared to the respective control group. The significant differences are marked with * for *P* < 0.05, and ** for *P* < 0.01. (**D**) H&E stains and immunohistochemical analyses of intestinal tissues from compound *Pygo2* deficient *Ctnnb1 GOF* mice, which were sacrificed on day 34 and day 86. Note that the intestine shows small areas with expression of PYGO2 (upper panel). These animals develop at later stages multiple microadenoma (lower panel) that express PYGO2, but not BCL9-2, with increased BrdU staining and concomitant nuclear ß-catenin. Scale bars in the pictures represent 200 μm for all IHC. Inserts show the staining at higher magnification.

First we compared the effects of *Pygo2* loss during the acute phase of intestinal tumor initiation induced by homozygous truncated *Apc (Apc* LOF; *Pygo2^+/+^, ^+/−^ and ^−/−^*) and by heterozygous stabilized ß-catenin (*Ctnnb1* GOF; *Pygo2*^+/+^, ^+/−^ and ^−/−^ ; Figures 2, 3). Loss of function of *Apc* led to a severe intestinal phenotype within one week post induction and all mutant mice died within seven to eight days. The survival of *Apc* mutant animals was not increased by the loss of one or both alleles of *Pygo2* Figure 2A). Analyses of intestinal tissues of mice sacrificed on day six after start of induction demonstrated massive intestinal hyperproliferation with elongated crypts, strong proliferation as detected by BrdU stain and upregulation of cytoplasmatic and nuclear ß-catenin (Figure 2B). However, knockout of *Pygo2* did not rescue the intestinal hyperproliferation induced by *Apc LOF.*

In contrast, introduction of one mutant *Ctnnb1* allele led to a less severe phenotype and all mice died between two and to seven weeks post induction (Figure 2C). Surprisingly, ablation of one allele of *Pygo2* prolonged the survival of *Ctnnb1 GOF* animals significantly (*n* = 36, *p* < 0.001; Figure 2C). Complete knockout of *Pygo2* prolonged the survival of *Ctnnb1* mutants in the initial phase for up to ten weeks (*n* = 37; *p* < 0.0001). We also analyzed the intestines of these compound mutant mice by immunohistochemistry. Intestinal hyperproliferation induced by *Ctnnb1 GOF* was mild starting on day six after induction and was most prominent on day 18, when compound *Ctnnb1 GOF*, *Pygo2* wild type animals started to die (Figure 2D and data not shown). The intestines of *Ctnnb1 GOF* mutant animals with PYGO2 wild type expression showed strong enlargement of crypts, increased proliferation and high levels of cytoplasmatic und nuclear ß-catenin. Remarkably, hyperproliferation was reduced in animals after ablation of one allele of *Pygo2*. Most notably, ablation of both *Pygo2* alleles completely rescued the phenotype of *Ctnnb1 GOF* animals: The intestinal architecture was similar to controls with normal crypt-villus axes, and proliferative cells were restricted to the stem cell zone (Figure 2D).

We next analyzed the expression of the *Pygo* and *Bcl9* cofactors by immunohistochemistry. Moreover, we enriched intestinal tissues for epithelial cells (see Material and Methods) and performed qRT-PCRs from the extracted epithelial RNA (Figure 3A–3C). PYGO2 was strongly expressed within the hyperproliferative cells of the intestine in both *Apc LOF* and *Ctnnb1 GOF* mutant animals with *Pygo2* wild type expression. On the RNA level, *Pygo2* was slightly increased only in *Ctnnb1 GOF* mice. Knockout of one *Pygo2* allele reduced RNA levels to approximately 50 percent. The complete knockout of both *Pygo2* alleles almost completely reduced *Pygo2* RNA levels, and PYGO2 protein was not detectable in the epithelial cells of the intestine, both in *Apc LOF* and *Ctnnb1 GOF* mutant mice. As expected, BCL9 expression remained unchanged in both mouse models. *Bcl9-2* transcription was not induced and the protein was completely absent in the hyperproliferative regions in *Apc LOF* mutants (Figure 3A, 3C). In *Ctnnb1 GOF-Pygo2* wild type mice, *Bcl9-2* was only slightly increased on the RNA level, but was not detectable on the protein level in the hyperproliferative cells (Figure 3B, 3C). These results provide *in vivo* evidence that *Bcl9-2* is not a target of deregulated Wnt/ß-catenin signaling during tumor initiation. However, transcription of *Axin2* as marker of activated Wnt signaling was strongly induced in both *Apc LOF* and *Ctnnb1 GOF* mutant animals (Figure 3C). Remarkably, *Axin2* was reduced in compound heterozygous and homozygous *Pygo2* deficient mice in both models. However, knockout of *Pygo2* completely reduced *Axin2* expression only in *Ctnnb1 GOF* mice to the control level, but not in *Apc LOF* animals. These findings further corroborate that *Pygo2* knockout completely rescued the initial phase of intestinal hyperproliferation induced by stabilized ß-catenin (*Ctnnb1*), but not of truncated *Apc*.

During the survival analyses of *Ctnnb1* mutant mice, we observed that homozygous *Pygo2* knockout animals survived the initial phase of tumor initiation for up to ten weeks after induction. However, these animals also died within four months (Figure 2C). We therefore analyzed the intestines of mutant animals by immunohistochemistry at later points of time. Approximately one month post induction, when all *Pygo2* wild type animals already died, we found small areas with increased proliferation and most importantly with re-expression of PYGO2 in single cells (Figure 3D upper panel). At later time points (approximately after three months; Figure 3D lower panel), the intestines of these mice were covered with multiple small adenoma that showed high proliferation and increased ß-catenin. PYGO2 was strongly expressed in these microadenoma, while the surrounding intestinal epithelial cells did not express PYGO2. Of note, these small adenoma did not express BCL9-2. These observations led us to hypothesize that these tumors may arise from single intestinal cells that may have escaped the initial recombination for the loss of *Pygo2*. Thus, re-expression of PYGO2 appears to promote microadenoma formation in compound *Ctnnb1 GOF*-*Pygo2* knockout animals at later stages.

We also analyzed in detail adenoma formation by the inducible loss of only one functional allele of *Apc*, which results in an *Apc^Min/+^* -like phenotype [25]. We compared animals that are wild type for *Pygo2* with hetero- and homozygous *Pygo2* deficient animals (Supplementary Figure S2). Survival and tumor development of animals was monitored and adenoma were analyzed by immunohistochemistry. Deletion of one or two alleles of *Pygo2* did not significantly change the overall survival of *Apc* mutant animals, which died between six and seven months post induction (Supplementary Figure S2A). Analyses of the intestines of mutant animals revealed that the tissues exhibited multiple small adenoma without any gross difference between *Pygo2* wild type and knockout (not shown). Loss of PYGO2 expression in the adenoma was confirmed by immunohistochemistry in *Pygo2* deficient animals. Moreover, all adenoma of the analyzed genotypes were highly proliferative, showed expression of nuclear ß-catenin and upregulation of BCL9-2 (Supplementary Figure S2B). Thus, *Pygo2* ablation apparently cannot prevent adenoma formation that is genetically induced by loss of one functional *Apc* allele *in vivo,* which also exhibits loss of the second allele due to loss-of-heterozygosity (LOH) [30, 31].

In summary, we provide *in vivo* evidence that *Pygo2* loss cannot compensate the intestinal hyperproliferation and adenoma formation resulting from *Apc* truncation. In contrast, *Pygo2* ablation rescues the initial phase of intestinal hyperproliferation that is induced by stabilized ß-catenin.

### *Pygo2* differentially activates key target genes during the initial phase of intestinal tumorigenesis in *Apc* versus *Ctnnb1* mutant mice and in chemically induced tumors

Next, we characterized the *in vivo* role of *Pygo2* for the transcriptional activation of Wnt/ß-catenin target genes and of genes that are implicated in intestinal tumor initiation and progression. We and others have previously shown that the nuclear co-factors of the *Bcl9* and *Pygopus* gene families are not general co-activators of canonical Wnt signaling, but that they activate a specific gene signature in normal and transformed cells [1, 8, 32–34]. Therefore, we hypothesized that *Pygo2* might also differentially activate such target genes *in vivo* during intestinal tumorigenesis.

**Figure 4:**
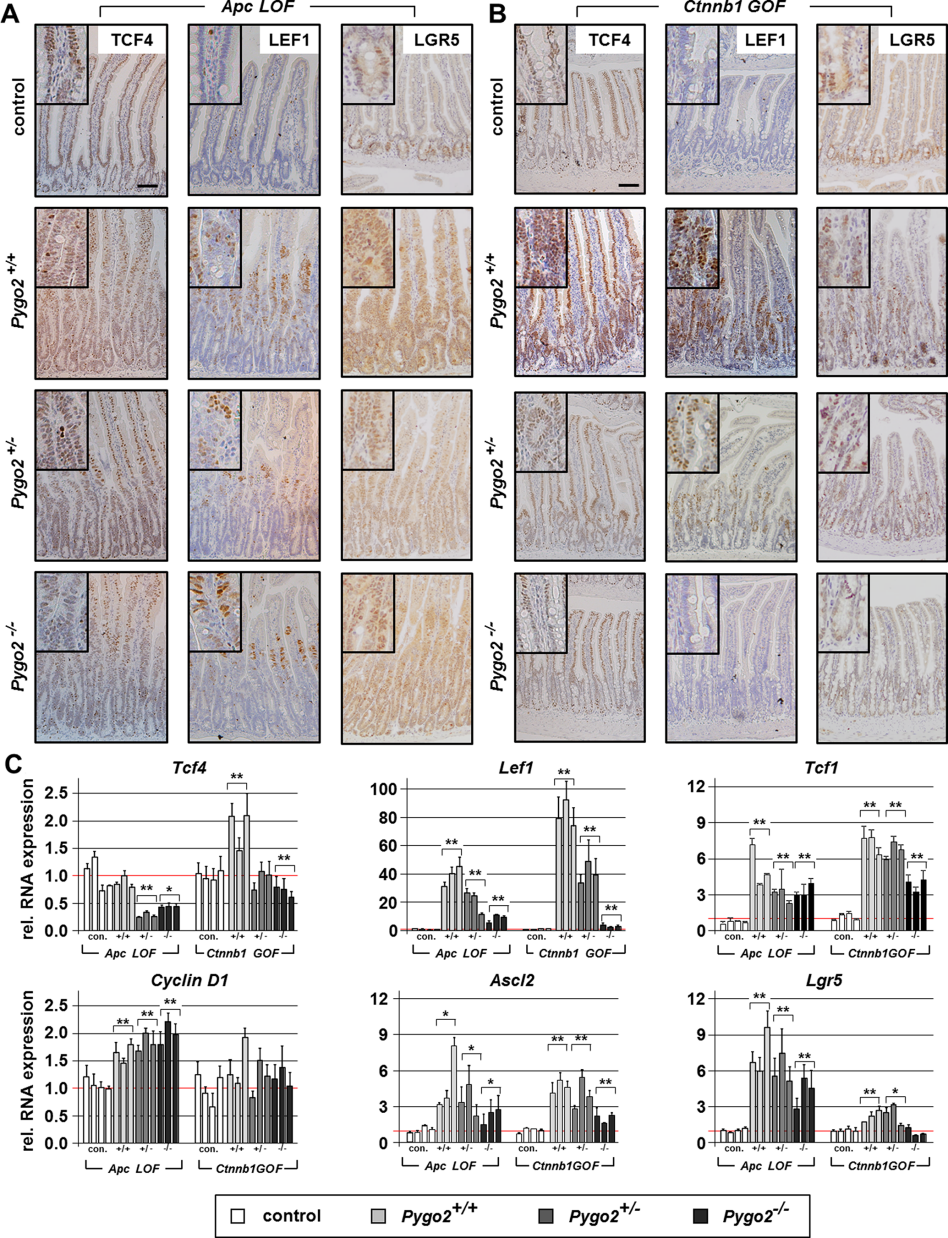
*Pygo2* ablation downregulates different Wnt/ß-catenin target genes overexpressed during intestinal hyperproliferation induced by *Apc LOF* and *Ctnnb1 GOF* (**A**, **B**) Representative Immunostains with the indicated antibodies on intestinal tissue sections from compound homozygous *Apc LOF* mice (A) and heterozygous *Ctnnb1 GOF* animals (B). The upper panel shows the stainings from controls. Pictures from immunostains of compound mutant animals with wild type *Pygo2* expression and hetero- and homozygous *Pygo2* ablation are provided below. Scale bars in the pictures represent 200 μm for all IHC. Inserts show the staining at higher magnification. (**C**) Relative RNA expression of Wnt/ß-catenin target genes in *Apc LOF* and *Ctnnb1 GOF* mutant animals. qRT-PCR was performed with RNA extracted from intestinal epithelial cells of compound mutant animals as indicated below the graphs. Significances are marked with *P < 0.05, and **P < 0.01 for the mean expression level compared to the respective control group.

First, we compared the initial phase of intestinal hyperproliferation induced by *Apc LOF* and *Ctnnb1 GOF* on day six and 18, respectively. We assessed the expression of the Lef/Tcf transcription factors, which are activated by Wnt/ß-catenin-signaling [35] (Figure 4A–4C and Supplementary Figure S3A–3B). In the normal intestine, TCF4 is expressed in the epithelial cells of the crypt compartment and along the crypt-villus axis with highest expression in the villi [36], which we also found in controls. During the initial phase of hyperproliferation, we established that *Tcf4* was not upregulated at the protein or RNA level in *Apc LOF* mice. *Tcf4* was moderately increased in *Ctnnb1 GOF* animals, both on protein and RNA expression levels. Remarkably, loss of one or two alleles of *Pygo2* downregulated *Tcf4* RNA in both mouse models, even below the expression level of controls. In contrast to *Tcf4*, we found that LEF1 protein was barely detectable in the normal intestine. *Lef1* protein and RNA levels were strongly upregulated, both in *Apc LOF* and *Ctnnb1 GOF* mice. Remarkably, elevated *Lef1* was strongly suppressed in *Pygo2* heterozygous and even stronger in homozygous knockout animals in both mouse models. However, *Lef1* was still higher in *Pygo2* deficient intestines of *Apc* and *Ctnnb1* mutant mice compared to controls. Full-length *Tcf1*, previously described as target of *Tcf4* [37], was also increased in *Apc* and *Ctnnb1* mutant tissues. However, ablation of *Pygo2* only moderately decreased overexpressed *Tcf1* levels. Taken together, *Pygo2* ablation reduces the overexpression of *Lef1* and *Tcf1,* and downregulates *Tcf4* in *Apc* mutant animals, but does not rescue intestinal hyperproliferation. In *Ctnnb1* mutants, however, the same repression of *Tcf1*, *Tcf4* and *Lef1* is seen after loss of *Pygo2*, but the intestinal hyperproliferation is completely rescued. Thus, the effect of *Pygo2* ablation on *Lef/Tcf* expression during the initial phase of tumorigenesis is apparently not linked to the outcome of the *Apc LOF* or *Ctnnb1 GOF* phenotype.

We further assessed the regulation of other known Wnt/ß-catenin target genes such as *Cyclin D1* (Figure 4C and Supplementary Figure S3A–3B). *Cyclin D1* transcription and protein expression was also increased in *Apc* mutant animals and remained unchanged by ablation of *Pygo2*. In contrast, *Cyclin D1* RNA was not increased but the protein was clearly expressed in the hyperproliferative cells in *Ctnnb1 GOF* animals. Again, *CyclinD1* protein expression was completely normal and restricted to the stem cell compartment in *Ctnnb1 GOF* animals with ablation of *Pygo2*, similar to controls. Moreover, we characterized the expression of the intestinal stem cell markers *Ascl2* and *Lgr5*, which are also Wnt/ß-catenin target genes in the cycling columnar cells at the crypt base [38–41] (Figure 4A–4C). As expected, the expression of both stem cell markers was increased in *Apc* and *Ctnnb1* mutant animals. *Lgr5* and *Ascl2* were highly increased in *Apc* mutant animals, and the overexpression was only slightly reduced by deletion of *Pygo2.* In contrast, ablation of *Pygo2* reduced upregulated *Ascl2* and completely suppressed the upregulation of *Lgr5* in *Ctnnb1 GOF* intestines to control levels. Of note, *Lgr5* was only moderately overexpressed in *Ctnnb1* mice compared to *Ascl2.* This observation may indicate that deletion of Pygo2 can reduce deregulated Wnt signaling to a certain threshold.

In summary, the overexpression of Wnt/ß-catenin target genes such as *Lef/Tcf's*, *Axin2*, *Cyclin D1, Ascl2* and *Lgr5* induced by truncated *Apc* cannot be rescued by loss of *Pygo2*. In contrast, ablation of *Pygo2* in the context of stabilized ß-catenin (*Ctnnb1*) counteracts the overexpression of these target genes. Moreover, these data suggest that the less severe phenotype of *Ctnnb1* mutant intestines is also linked to lower levels of increased Wnt/ß-catenin target gene expression. Thus, knockout of *Pygo2* is apparently sufficient to reduce target gene expression to normal levels in this model. In summary, *Pygo2* loss apparently reduces deregulated Wnt signaling gene output to a certain threshold.

**Figure 5:**
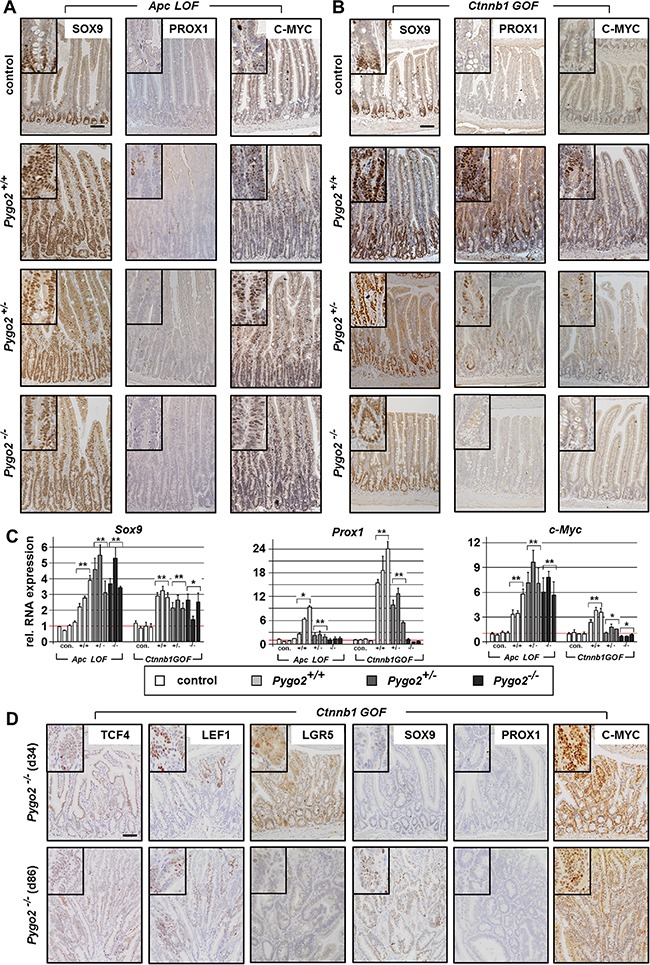
*Pygo2* ablation completely suppresses the upregulation of Prox1, but fails to decrease the overexpression of c-Myc during early intestinal tumorigenesis in *Apc LOF* mice (**A**, **B**) Immunostains for detecting tumor progression markers SOX9, PROX1 and C-MYC on intestinal tissue sections of compound *Apc LOF* (A) and *Ctnnb1 GOF* animals (B). (**C**) qRT-PCR analyses for the relative RNA expression of *Sox9, Prox1 and C-myc*. Each bar represents one animal. Each graph represents the mean of at least three independent experiments from RNA of mice with the indicated genotype. Significant difference for the mean expression level relative to the corresponding control group is marked * for *P* < 0.05, and ** for *P* < 0.01. (**D**) Immunohistochemical analyses of intestinal tissues from compound *Pygo2* deficient *Ctnnb1 GOF* mice on day 34 and day 86 post induction. Sections were stained with indicated antibodies for detecting Wnt/ß-catenin targets and tumor progression markers. Note that microadenoma also do not express PROX1, but show overexpression of C-MYC in the adenomatous cells. Scale bars in the pictures represent 200 μm for all IHC. Inserts show the staining at higher magnification.

Next, we characterized the importance of *Pygo2* for the expression of differentiation markers in *Apc* and ß-catenin *(Ctnnb1)* mutant intestines (Figure 5A– 5C, Supplementary Figure S3C–3E). Paneth cells located in the crypt bottom are characterized by the expression of Lysozyme and of the Wnt/ß-catenin target gene *Sox9* [42, 43]. Following *Apc* induced intestinal hyperproliferation we found Paneth-like cells throughout the hyperproliferative epithelium as indicated by Lysozyme stains. Moreover, *Sox9* RNA was strongly induced and the protein was almost completely expressed in all cells along the crypt-villus axis. These *Apc* induced changes of *Sox9* expression and cell differentiation were unchanged by ablation of *Pygo2*. Overexpression of *Sox9* and mislocalization of Paneth-like cells were also present in *Ctnnb1 GOF* intestine, although less pronounced. Knockout of *Pygo2* only partially rescued these changes. In particular, SOX9 was still moderately overexpressed (Figure 5A–5C, Supplementary Figure S3C–3D). Thus, loss of *Pygo2* has no or little effect on the generation of Paneth-like cells in the hyperproliferative intestine of *Apc* or *Ctnnb1* mutants.

We also studied the expression of *Dclk1* that marks Tuft cells in the normal intestine and was recently suggested to be a tumor stem cell marker in the intestine [44] (Supplementary Figure S3C–3E). *Dclk1* expression was unchanged in *Ctnnb1 GOF* mutants and not affected by *Pygo2* loss. Remarkably, *Dclk1* was completely lost in *Apc LOF* mutants, both on Pygo2 wild type and knockout background. This might reflect the differentiation defects due to relatively severe hyperproliferation in *Apc* mutants.

Finally, we characterized transcription factors, which are important for tumor progression during intestinal carcinogenesis. We analyzed *Prox1*, a stem cell regulator in intestinal tumors that promotes the transition from benign to a malignant phenotype by oncogenic Wnt/ß-catenin signaling (Figure 5A–5C) [45, 46]. In fact, we found strong upregulation of *Prox1* in *Apc LOF* and even more prominent in *Ctnnb1 GOF* mice. Most interestingly, *Pygo2* knockout completely downregulated *Prox1* levels to control levels in both animal models. However, despite the downregulation of *Prox1* in *Pygo2* deficient animals, the hyperproliferation induced by mutant *Apc* was not rescued. These data suggest that *Pygo2* function on *Prox1* expression is not essential for intestinal tumorigenesis during early stages in our models.

Last, we analyzed *c-Myc*, a key transcription factor for early stages of intestinal tumorigenesis that is activated by Wnt/ß-catenin signaling [47] (Figure 5A–5C). Of note, genetic deletion of *c-Myc* was shown to completely rescue the phenotype of *Apc* induced early tumorigenesis [48]. In fact, we found strong upregulation of *c-Myc* RNA levels and protein expression in *Apc LOF* mice. However, knockout of *Pygo2* failed to downregulate *c-Myc* overexpression in *Apc* mutant animals. Similarly, *c-Myc* was also upregulated in *Ctnnb1 GOF* intestines however, this was less pronounced compared to *Apc* mutants. Importantly, deletion of one *Pygo2* allele downregulated *c-Myc* and knockout of both *Pygo2* alleles completely suppressed *c-Myc* expression even below control levels. Thus, the function of *Pygo2* for hyperactivated Wnt/ß-catenin signaling appears to increase the signaling output to a certain threshold. Overexpressed *c-Myc* may represent a key target, which can be downregulated to a certain threshold by *Pygo2* deletion during tumorigenesis.

We also analyzed the target genes in microadenoma of *Pygo2* deficient-*Ctnnb1 GOF* mice within three months after induction (Figure 5D and data not shown). These tumors emerged from *Pygo2* escapers (see Figure 3D) and demonstrated also overexpression of TCF4, LEF1 and SOX9, but still lacked PROX1. Moreover, LGR5 was only slightly increased within the adenomatous cells. Most importantly, the microadenoma showed also strong expression of C-MYC, which further supports that C-MYC expression was apparently activated by *Pygo2* in *Ctnnb1 GOF* mutants.

Of particular note, intestinal epithelial specific conditional loss of *Pygo2* in mice did not influence the expression of most of the Wnt/ß-catenin target genes analyzed in our study (Supplementary Figure S4). Only the relative RNA expression of *Tcf4* and *Lef1* was slightly, but significantly, downregulated compared to controls. However, these minor changes in gene expression are apparently not sufficient to result in any phenotypic changes. These data further suggest that *Pygo2* is apparently dispensable for the expression of target genes in the normal intestine.

Recently, *Pygo2* was also implicated in Wnt/ß-catenin-independent functions, for instance to increase Notch signaling in mammary gland cells [49]. We therefore asked if there might be a similar effect in *Pygo2* deficient intestines in our animal models (Supplementary Figures S3E, S4 and S5B). We analyzed the RNA levels of *Hes1* as the most abundantly expressed Notch target gene in the intestine [55]. *Hes1* is expressed in proliferating crypt cells that differentiate to absorptive enterocytes [50]. However, *Hes1* RNA was not upregulated in the hyperproliferative intestine of both *Apc LOF* and *Ctnnb1 GOF* mice and in chemically induced tumors. Genetic ablation of *Pygo2* did only slightly downregulate *Hes1* transcription in *Ctnnb1 GOF* animals, but not in *Apc LOF* mice and in chemically induced tumors. Moreover, *Hes1* was also not suppressed in the normal intestine by loss of *Pygo2*. This may suggest that Notch signaling is not a major target of *Pygo2* in the intestine.

**Figure 6:**
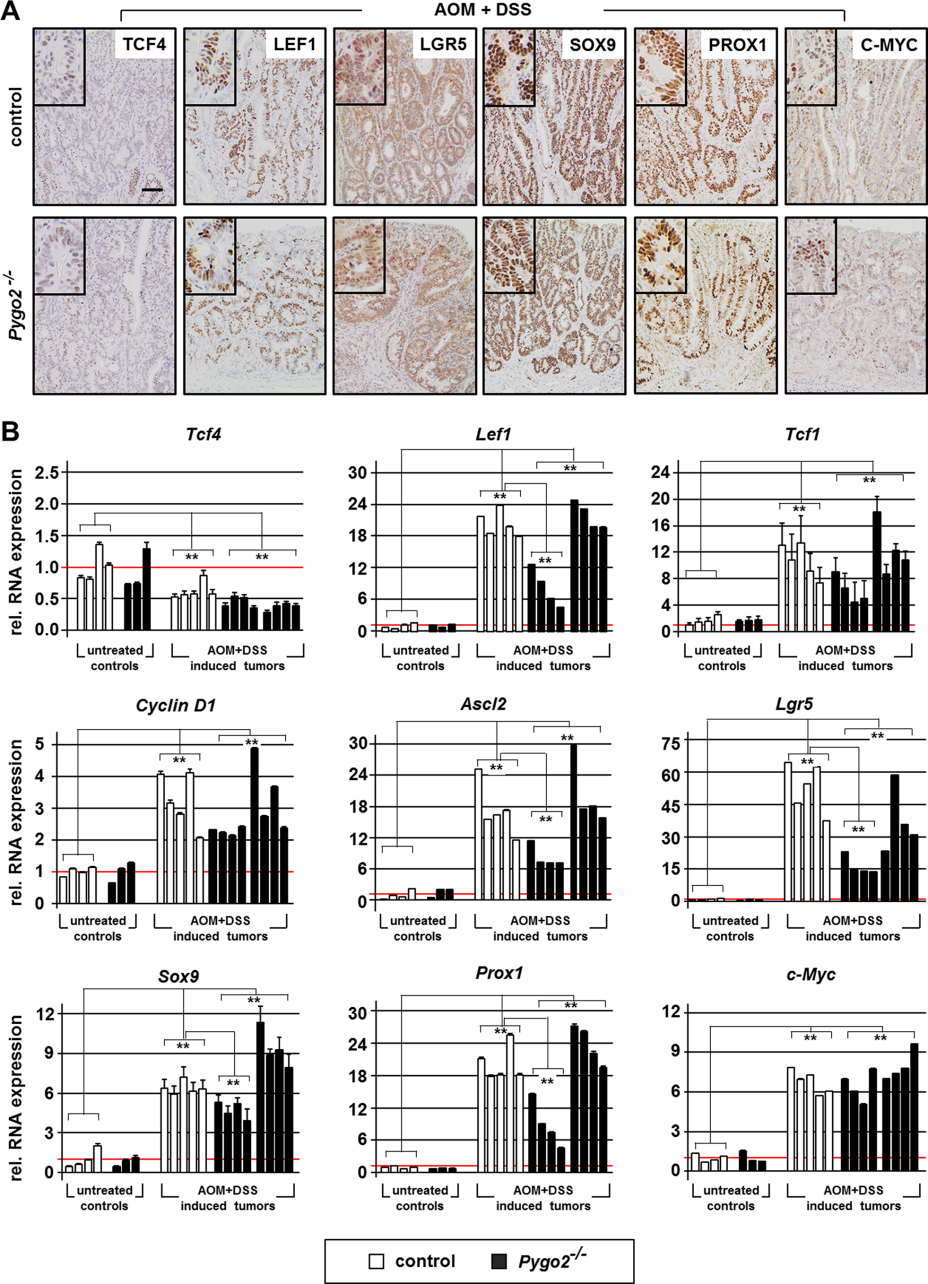
Knockout of *Pygo2* downregulates overexpressed Wnt/ß-catenin targets and tumor progression genes in a subgroup of *Pygo2* deficient chemically induced intestinal tumors (**A**) Representative immunohistochemical stainings of chemically induced intestinal tumors for the indicated markers. Tissues from controls (*Pygo2^lox(ex3)/lox(ex3)^*) and *Pygo2* knockout animals (*Vil^Cre^; Pygo2^Δ/Δ^*) were collected six months after treatment with AOM and DSS. Scale bars in the pictures represent 200 μm for all IHC. Inserts show the staining at higher magnification. (**B**) qRT-PCR analyses of RNA extracted from tumors and from colon tissues of untreated mice, both of controls and *Pygo2* deficient animals. Each graph shows the relative RNA expression of the indicated gene for one single animal. Significances were calculated for the mean expression level compared to untreated controls and relative to the expression levels of control tumors, respectively. ** marks significant differences between the respective groups with *P* < 0.01.

Finally, we analyzed if these target genes were also affected in chemically induced intestinal tumors following *Pygo2* knockout. We analyzed the expression by immunohistochemistry and qRT-PCR on RNA extracted from the tumors (Figure 6A, 6B and Supplementary Figure S5). In fact, all target genes with the exception of *Tcf4* were also strongly overexpressed in the tumors of control and *Pygo2* deficient animals, both on the protein and RNA level. However, a subgroup of chemically induced tumors derived from *Pygo2* deficient animals showed also significant reduction of the overexpression of *Lef1, Ascl2, Lgr5, Sox9* and *Prox1* RNA levels compared to control tumors (Figure 6B). In contrast and most importantly, the transcription of *c-Myc* was not downregulated by *Pygo2* ablation. Thus, also chemically induced intestinal tumors show a similar pattern of gene regulation in the context of *Pygo2* knockout as seen in early stages of tumorigenesis induced by *Apc LOF* or *Ctnnb1 GOF*. Moreover, deletion of *Pygo2* cannot rescue the overexpression of *c-Myc* in this tumor model.

Taken together, we have identified molecular targets that are differentially activated by *Pygo2* during intestinal tumorigenesis in our mouse models. *Pygo2* ablation reduces the overexpression of several key Wnt/ß-catenin target genes and apparently reduces the signaling threshold. In particular, the overexpression of *c-Myc* is not rescued by *Pygo2* knockout in *Apc* LOF tissues and in chemically induced tumors. This might explain why *Pygo2* knockout cannot prevent tumorigenesis, but reduces tumor formation *in vivo*.

## DISCUSSION

*Pygopus* was discovered as Wnt signaling co-activator important for embryonic development in Drosophila [51]. Since this discovery, the role of the vertebrate homologues *Pygo1* and *Pygo2* has been studied and it was shown that they exert a limited role during Wnt/ß-catenin dependent embryonic development [52, 53]. Recent studies focused on their oncogenic role in the context of deregulated Wnt signaling during cancer development [54, 55]. We have previously shown that *Pygo2,* but not *Pygo1,* is overexpressed in different stages of human colon cancer and in adenoma of APC^Min/+^ mice [8]. In the present study, we have analyzed the *in vivo* role of *Pygo2* using chemically induced colon cancer and conditional *Apc* LOF and *Ctnnb1* GOF mouse models. Intriguingly, we found that *Pygo2* deletion delayed chemically induced colon tumor formation and totally suppressed intestinal hyperproliferation induced by stabilized ß-catenin. In contrast, *Pygo2* knockout failed to suppress intestinal hyperproliferation and adenoma formation in *Apc* loss of function driven intestinal tumorigenesis. We hypothesized that this different effect may critically be linked to varying dosages of Wnt signaling output that are activated by *Pygo2.* Based on our results, we indeed found that several genes were differentially suppressed by *Pygo2* knockout in our tumor models.

We confirmed in our chemically induced colon tumor model that Wnt signaling is in fact overactivated. This was indicated by elevated nuclear ß-catenin and overexpression of *Axin2* in the tumors. These results are in line with previous reports that described hyperactive Wnt signaling and mutations of the *Apc* or *Ctnnb1* genes in this tumor model [56, 57]. Most importantly, *Pygo2* deletion significantly reduced tumor number and size. Although we did not see any apparent difference in tumor invasiveness or angiogenesis between controls and knockout animals, we suggest that *Pygo2* is involved in tumor progression with respect to growth of the primary tumor. Similar to our study, delayed tumor development following chemical induction was also observed in *Bcl9/Bcl9-2* double mutants [9]. Of note, these co-activators are also dispensable for normal intestinal homeostasis similar to *Pygo2* [58]. Besides deregulated Wnt-signaling, chemically induced tumors have been shown to harbor additional mutations in other pathways such as *TGF-ß* and *K-ras* signaling [27, 59]. It is therefore possible that *Pygo2* loss and the effects on Wnt signaling are not sufficient to completely suppress colon tumor formation in this animal model. However, our data indicate that *Pygo2* deletion delays tumor growth by inhibition of Wnt signaling in chemically induced colon tumors. Together, these data suggest that the Wnt signaling co-activators of the *Bcl9* and *Pygopus* family are apparently dispensable for normal intestinal homeostasis. In contrast, *Pygo2*, similar to *Bcl9*/*Bcl9-2*, may drive intestinal tumor formation by increasing aberrant Wnt signaling *in vivo*.

Our findings regarding the phenotype of homozygous *Apc LOF* and heterozygous *Ctnnb1 GOF* mutants with *Pygo2* wildtype background are in accordance with previous reports [24, 25, 48]. Mice homozygous for *Apc* LOF showed enlarged crypt-villus axes within few days, indicating massive intestinal hyperproliferation, and survived only seven to eight days after induction. The phenotype was less severe in heterozygous *Ctnnb1* mutants with intestinal hyperproliferation starting after two weeks and the overall survival of *Pygo2* wildtype animals was less than three weeks. Most importantly, *Pygo2* ablation completely rescued the intestinal phenotype in heterozygous *Ctnnb1* GOF mutants, but not in *Apc* LOF mutants. Although *Pygo2* knockout in *Ctnnb1 GOF* mice completely rescued the initial intestinal phenotype, these mice however also died several weeks later than Pygo2 wild type animals. Remarkably, we found PYGO2 re-expression in single intestinal cells of knockout animals. This is most likely due to failed recombination of *Pygo2* deletion as reported for other knockout models [60]. These *Pygo2* “escaper” cells induced apparently microadenoma, which led to a lethal phenotype of *Pygo2* knockout-*Ctnnb1 GOF* mice at much later points of time. This is a further *in vivo* evidence for the crucial role of *Pygo2* during intestinal tumorigenesis induced by *Ctnnb1 GOF*. Thus, complete loss of *Pygo2* may in fact extend the life span of *Ctnnb1 GOF* mice that is comparable to normal mice.

We also studied the phenotype of heterozygous *Apc LOF* mice. These mice exhibited an *Apc^Min/+^*-like phenotype with development of multiple small intestinal adenoma [61]. In this tumor model, the second functional *Apc* allele is lost over time in single epithelial cells (loss of heterozygosity, LOH), which induces adenoma formation[31]. In fact, we found upregulation of nuclear ß-catenin and overexpression of *Pygo2* in the adenoma. However, deletion of *Pygo2* did also not rescue adenoma formation in this *Apc* LOF model. Thus, *Pygo2* deletion is apparently not sufficient to re-establish normal levels of Wnt signaling and to prevent tumorigenesis in either of the *Apc LOF* mutants studied here, in contrast to *Ctnnb1 GOF* mutants.

The overall persistence of the phenotype in *Apc LOF* animals despite *Pygo2* knockout may be linked to additional Wnt-independent functions of *Apc*. Among these, *Apc* is crucial for chromosome segregation, migration, apoptosis and differentiation [62, 63]. In addition, it was shown that loss of *Apc* drives chromosomal instability in adenoma of *Apc* mutants and in chemically induced colon tumors of mice [64, 65]. Thus, *Pygo2* loss might not have compensated these Wnt-independent functions of *Apc.* Indeed, a recent study revealed that restoration of APC wild type protein rescued intestinal tumorigenesis in mice even in the presence of *K-ras* and *TP53* mutations [66].

We have analyzed in our tumor models the expression of all four members of the *Pyogous* and *Bcl9* co-factor families (summarized in Table 1). We found in all animal models induction of PYGO2 expression, but not of PYGO1. PYGO2 was overexpressed in the hyperproliferative regions during early tumorigenesis, but also in adenoma and colon tumors in mice with *Pygo2* wild type background. These results are in accordance with our previous studies in APC^Min/+^ mice, human colon cancers and colon cancer cell lines [8]. In *Pygo2* knock out mice, PYGO2 was clearly downregulated to undetectable levels in all tumor models. Of note, we did not see compensatory upregulation of PYGO1 in *Pygo2* knockout tissues. In fact, we have previously not detected PYGO1 expression in the normal intestine and during different stages of tumorigenesis in *Apc^Min/+^* tumors and human colon cancer cells (this study and [8]). Therefore, it appears unlikely that *Pygo1* may have compensated the intestinal phenotype of *Pygo2* knockout animals. BCL9 expression was also not induced in our tumor models, similar to our previous studies [8]. In contrast, BCL9-2 was expressed in chemically induced tumors, and strongly overexpressed in adenoma of heterozygous *Apc LOF* mice. In contrast, BCL9-2 was not induced in the hyperproliferative regions of homozygous *Apc LOF* and only slightly increased in heterozygous *Ctnnb1 GOF* mice. Thus, BCL9-2 overexpression appears to be linked to more advanced stages of tumorigenesis, as we found in APC^Min/+^ mice and human colon cancers [8]. Taken together, current results support our previous results, that BCL9 and BCL9-2 are not targets of active Wnt/ß-catenin signaling, in contrast to other studies [15]. Thus, BCL9-2 overexpression in advanced stages of tumorigenesis might be linked to additional genetic events such as genomic instability leading to overexpression of the gene as previously found for BCL9 [67]. Moreover, upregulation of BCL9-2 in tumors and adenoma was unchanged by *Pygo2* ablation, indicating that *Bcl9-2* expression is not activated by *Pygo2*. In fact, BCL9-2 expression is apparently suppressed by active Wnt signaling as we found loss of BCL9-2 expression in the hyperproliferative regions in both *Apc* and *Ctnnb1* mutant models. This is in line with our finding, that BCL9-2 is absent in the crypts of the normal epithelium where Wnt signaling is active [8]. Moreover, ß-catenin negatively regulated the expression of *Bcl9-2* in human colon cancer cells [8]. Thus, we speculate that the absence of BCL9-2 in the hyperproliferative regions might be a direct or indirect effect of active Wnt/ß-catenin signaling. Overall, *Bcl9-2* upregulation in adenoma of heterozygous *Apc* mice and chemically induced tumors may further drive tumor progression, which cannot be compensated by loss of *Pygo2* [8, 14, 16]. However, the exact molecular mechanism for the regulation of the *Pygo* and *Bcl9* co-factors and their role in *Apc*- or *Ctnnb1*-associated colorectal cancer requires further analyses.

**Table 1:** Summary for the relative regulation of target gene expression in the tumor models with and without knockout of Pygo2

tumor model	AOM+DSS tumors		Apc LOF		Ctnnb1 GOF	
genotype	Pygo2 WT	Pygo2 KO	Pygo2 WT	Pygo2 KO	Pygo2 WT	Pygo2 KO
**Wnt/ß-catenin signaling co-factors**						
***Pygo2***	**↑**	**↓↓↓**	**↑**	**↓↓↓**	**↑↑**	**↓↓↓**
***Pygo1***	**n.e.**	**n.e.**	**n.e.**	**n.e.**	**n.e.**	**n.e.**
***Bcl9***	**≈**	**≈**	**≈**	**≈**	**≈**	**≈**
***Bcl9-2***	**↑↑**	**↑↑**	**≈**	**≈**	**(↑)**	**≈**
**Classical Wnt target genes**						
***Axin2***	**↑↑↑**	**↑**	**↑↑**	**↑**	**↑↑**	**≈**
***CyclinD1***	**↑↑**	**↑↑**	**↑↑**	**↑↑**	**↑**	**≈**
***Tcf1***	**↑↑↑**	**↑↑**	**↑↑↑**	**↑↑**	**↑↑↑**	**↑↑**
***Tcf4***	**≈**	**↓**	**≈**	**↓**	**↑**	**↓**
***Lef1***	**↑↑↑**	**↑**	**↑↑↑**	**↑**	**↑↑↑**	**↑**
**Stem cell marker**						
***Ascl2***	**↑↑↑**	**↑↑**	**↑↑↑**	**↑↑**	**↑↑**	**↑**
***Lgr5***	**↑↑↑**	**↑↑**	**↑↑↑**	**↑↑**	**↑**	**≈**
***Dclk1***	**↓**	**↓**	**↓**	**↓**	**≈**	**≈**
**Tumor progression marker**						
***Sox9***	**↑↑↑**	**↑↑**	**↑↑↑**	**↑↑↑**	**↑↑**	**↑**
***Prox1***	**↑↑↑**	**↑↑**	**↑↑**	**≈**	**↑↑↑**	**≈**
***c-myc***	**↑↑**	**↑↑**	**↑↑↑**	**↑↑↑**	**↑ / ↑↑**	**≈**

Relative expression level of target genes analyzed in this study. The summary is based on RNA expression as determined by qRT-PCR analyses and protein expression as detected by IHC. The increase ↑ or decrease ↓ is indicated relative to the respective uninduced controls in normal intestinal tissues. ≈ marks unchanged expression compared to control. Shown are the results from chemically induced tumors (AOM+DSS tumors) on *Pygo2* wild type background and the subgroup of tumors with reduced expression following *Pygo2* ablation (left column). The middle and right columns summarize the relative expression in homozygous *Apc* LOF and heterozygous *Ctnnb1* GOF mutants with *Pygo2* wild type expression compared to *Pygo2* knockout animals.

Our data suggested that *Pygo2* knockout might have rescued the intestinal phenotype in *Ctnnb1 GOF* mutants by suppressing specific Wnt target genes that are overexpressed during tumorigenesis. We speculate that such genes might be strongly deregulated in the early phase of *Apc* LOF animals and chemically induced tumors, which cannot be compensated by *Pygo2* loss to rescue the phenotype. This hypothesis was first confirmed in *Ctnnb1* GOF mice by the complete downregulation of increased *Axin2*, the best marker of active Wnt signaling [68]. In contrast, *Axin2* was strongly overexpressed in *APC* LOF mutants and in chemically induced tumors, and only partially suppressed by *Pygo2* knockout. We further analyzed several classical Wnt target genes and genes, which have been shown to be important for intestinal tumorigenesis. Our results provide several remarkable observations in the tumor models studied here with respect to their described role during tumorigenesis. We have summarized our results based on RNA and protein expression studied by qRT-PCR and IHC respectively in Table 1.

The overexpression of *CyclinD1,* another classical Wnt target gene that is deregulated during tumorigenesis [69], was suppressed by *Pygo2* knockout, similar to *Axin2*. *CyclinD1* was strongly upregulated in chemically induced tumors and *Apc* LOF mutants as previously described [70, 71]. Overexpressed *CyclinD1* however was only partially downregulated by *Pygo2* loss in these two animal models. In contrast, *CyclinD1* was only moderately increased in *Ctnnb1* mutants and completely reduced to control levels in *Pygo2* deficient animals.

The different members of the *Lef/Tcf* transcription factor family have been implicated in tumorigenesis[72], and we found strong upregulation of *Tcf1* and *Lef1* in the tumor models, which were only in part downregulated by *Pygo2* loss*. Tcf4* was only induced in *Ctnnb1* mutants, and the expression was downregulated by *Pygo2* knockout. Remarkably, *Tcf4* was not increased in chemically induced tumors and *Apc* LOF mutants, and was suppressed even below control levels in *Pygo2* deficient animals. In fact, *Tcf4* might represent a tumor suppressor as previously suggested by studies with *Tcf4* knockout animals that developed hyperproliferation in the colon [73]. Another study suggested that *Tcf4* appears to be important for intestinal homeostasis [74]. Of note, we found that the expression of *Tcf4* was suppressed by *Pygo2* loss in normal intestinal cells, indicating that *Tcf4* is a *Pygo2* dependent Wnt-target gene. However, since the *Pygo2* knockout in the intestine has no influence on intestinal homeostasis (Schelp and Brembeck, unpublished data), it is most likely that *Pygo2* is not essential for *Tcf4* expression due to the lack of an apparent phenotype.

We characterized intestinal stem cell markers that are activated by Wnt/ß-catenin signaling. *Lgr5* and *Ascl2* are crypt stem cell markers expressed in the cycling stem cells of the crypt[39, 41]. In line with human intestinal cancers [38, 75], *Lgr5* and *Ascl2* were overexpressed in all three tumor models indicating an increased number of stem-like cells as a result of aberrant Wnt signaling [76, 77]. *Ascl2* was strongly induced in all animal models and partially downregulated by *Pygo2* knockout*. Lgr5* was moderately induced in *Ctnnb1* GOF mice and ablation of *Pygo2* resulted in complete downregulation of *Lgr5*. In contrast, the strong overexpression of *Lgr5* in chemically induced tumors and in *Apc* LOF mice was only partially downregulated by *Pygo2* ablation. This may indicate that the tumor promoting function of *Pygo2* in the *Ctnnb1 GOF* model may be partly regulated by the induction of *Lgr5*-positive stem-like cells that are known to be the origin of intestinal cancer [78].

Recently, *Dclk1* was suggested as putative cancer stem cell marker in the intestine since *Dclk1* ablation induced the regression of adenoma in *APC^Min/+^* mice [44]. *Dclk1* is normally expressed in tuft cells of the intestine [44]. However, *Dclk1* was completely lost in chemically induced tumors and in *Apc LOF* mice and unchanged in *Ctnnb1* GOF mutants. Moreover, *Pygo2* ablation did not change *Dclk1* expression. The loss of *Dclk1* in our tumor models might reflect the disturbed differentiation in the colon tumors and the relatively high Wnt signaling level in *Apc* LOF mice [44]. In fact, a recent study showed that *Dclk1* is silenced in human colon cancer by promoter methylation [79]. Therefore, further studies are required to assess the reliability of *Dclk1* as a cancer stem cell marker.

Finally, we addressed the expression of known tumor progression markers, which have been implicated in intestinal tumorigenesis. *Sox9*, a target of Wnt signaling expressed in Paneth cells, was shown to be overexpressed in several human malignancies including colon cancer [80, 81]. We also found *Sox9* overexpression in chemically induced colon tumors and in *Apc* and *Ctnnb1* mutants. *Pygo2* failed to suppress *Sox9* overexpression in *Apc LOF*, and only partially reduced elevated Sox9 in colon tumors and *Ctnnb1* GOF mice. The transcription factor *Prox1,* another Wnt target gene, was characterized as an oncogene: *Prox1* ablation inhibited, and overexpression promoted tumor formation in *Apc^Min/+^* mice, respectively [45, 46]. Remarkably, overexpressed *Prox1* in the early stage of intestinal hyperproliferation in *Apc* LOF and *Ctnnb1* GOF mutants was completely downregulated to control levels in *Pygo2* deficient animals. Thus, despite the complete downregulation of *Prox1*, *Pygo2* ablation did not rescue the phenotype of *Apc* mutants, indicating that *Prox1* overexpression is not important for intestinal hyperproliferation. Moreover, *Prox1* was strongly overexpressed in chemically induced tumors, and partially reduced by *Pygo2* ablation. Therefore, *Prox1* is apparently a Wnt target gene that requires the co-activator function of *Pygo2*, but is not essential for early intestinal tumorigenesis in our models. The most important finding for the role of *Pygo2* in our study was the regulation of *c-Myc* as an essential transcription factor for colon cancer [82]. The strong *c-Myc* overexpression in chemically induced tumors and in *Apc* LOF animals was unchanged by *Pygo2* ablation. In contrast, the moderate overexpression of *c-Myc* in *Ctnnb1 GOF* animals was completely reduced to control levels. Of note, previous studies have shown that knockout of *c-Myc* rescued tumorigenesis in *Apc* LOF mice [48, 83]. Thus, *Pygo2* loss may have rescued the intestinal hyperproliferation of *Ctnnb1 GOF* mice predominantly by downregulating *c-Myc* representing a global gene amplifier [84]. Therefore, we propose that the role of *Pygo2* in intestinal tumorigenesis is dependent on the Wnt signaling dosage and the deregulation of specific target genes. Different dosages of Wnt signaling output have been studied in the context of embryonic stem cell differentiation and also for intestinal tumorigenesis [85–87]. In particular, specific *Apc* mutations induce different Wnt signaling dosages and thus determine the susceptibility for intestinal tumorigenesis [85]. Moreover, similar to the effects in our study in *Ctnnb1 GOF,* it was recently shown that *Pygo2* loss suppressed skin hyperplasia induced by stabilized ß-catenin *in vivo* [88]. This further underscores the importance of *Pygo2* for the deregulated Wnt signaling output induced by mutant ß-catenin. Further studies are required to elucidate the underlying molecular mechanism.

Altogether, our study highlights that deregulated Wnt signaling induced by stabilized ß-catenin can be repressed by *Pygo2* loss. This function may require a certain level of hyperactive Wnt signaling. Thus, targeting *Pygo2* in malignant tumors that harbor *Ctnnb1* mutations may result in suppression of tumor formation and growth. Interestingly, several human cancers have been characterized with ß-catenin mutations at higher frequencies compared to colon cancer. Among these tumors are hepatocellular carcinomas (HCC), solid-pseudopapillary tumors of the pancreas and almost all ovarian solid pseudopapillary tumors [89–91]. Therefore, small molecules that inhibit the interaction of PYGO2 with ß-catenin via BCL9/BCL9-2 may be of therapeutic value. In fact, Pyrvinium pamoate, an anti-helminth drug, was shown to decrease PYGO2 protein levels in colon cancer cells lines with *Apc* and *Ctnnb1* mutations [92], and in *Apc^Min/+^* mice [93]. However, since this drug apparently has also toxic effects, further compounds have to be developed and tested *in vivo* to target *Pygo2* for the treatment of cancers with mutant ß-catenin. This is an interesting therapeutic strategy, since targeting *Pygo2* in normal cells apparently will not disturb tissue homeostasis.

## MATERIALS AND METHODS

### Animal maintenance

All animal experiments were performed in accordance with German guidelines (TierSchG) and approved by governmental authorities. Animals were maintained on a pure C57BL/6 background. In all experiments, age-matched, non-transgenic littermates were used as controls. All mouse strains were grown and bred in European Neuroscience Institute, and Animal facility in University Medical Center, Göttingen.

### Animal models

The generation of ß-catenin conditional mutants was previously described [24]. In brief, *Ctnnb1* GOF animals used in our study harbor one mutant beta-catenin allele with loxP sites flanking exon 3 (*Ctnnb1^lox(ex3)/+^)*. This results after conditional recombination in deletion of 76 amino acids encoding the ß-catenin phosphorylation sites (*Ctnnb1^Δex3/+^)*. Conditional *Ctnnb1* GOF mice express a truncated mutant ß-catenin, which cannot be degraded, and therefore results in aberrant Wnt signaling activation [24].

*Apc* LOF animals were conditionally generated with mice harboring *Apc* mutant alleles with loxP sites flanking exon 15 (*Apc^lox(ex15)/lox(ex15)^*; [25]. After recombination, homozygous mutant mice express truncated APC protein lacking almost all functional APC domains that results in hyperactivated Wnt signaling (*Apc^Δex15/Δex15^*; [25]. In addition, heterozygous *Apc* mutant mice were generated (*Apc^Δex15/+^)*, which develop multiple intestinal adenoma after loss of the second allele as previously described [25].

The conditional *Pygo2* knockout was generated by insertion of the loxP-flanked deletion cassette in exon 3 that encodes the essential NHD and PHD domains of the PYGO2 protein *(Pygo2^lox(ex3)/lox(ex3)^*; H.J. Schäffer and W. Birchmeier, unpublished) and results in a null-phenotype after recombination. Details on generation of mice and phenotype will be described elsewhere. In our study*, Pygo2* mutant mice were generated by mating hetero- or homozygous floxed mice with intestine specific transgenic Cre-mice (*Pygo2^Δ/+^ and Pygo2 ^Δ/Δ^*). For induction of recombination, we used constitutive Vil-Cre and inducible Vil-CreERT transgenic mice that were previously described (*Vil^Cre^; Vil^Cre-ERT^*[29].To induce conditional intestine specific recombination, six- to eight-weeks old mice were injected intraperitoneally with 1 mg tamoxifen per mouse for five continuous days. All *Apc* LOF homozygous mice were sacrificed on day six. *Apc* LOF heterozygous mice were sacrificed on day 70. ß-catenin (*Ctnnb1 GOF*) heterozygous mice were sacrificed on day 6 and 18. Compound Pygo2 knockout *Ctnnb1* GOF animals were sacrificed in addition on day 30, 34 and 86, respectively. During the survival study, all mice were monitored daily to record the date of death. In all experiments, uninduced Cre-negative littermates with the respective genotype were used as controls.

### AOM and DSS treatment

For the regeneration studies dextran sodium sulphate (DSS, MP Bio medicals, 216011050) was orally administered. Six- to eight-weeks old mice (control and *Pygo2*^−/−^) were fed with 1.5% (w/v) DSS in drinking water for five consecutive days. Mice were monitored regularly for the health status and sacrificed on day 14 and day 28 for the histological examinations. For chemically induced colon tumorigenesis, a single dose of 10 μg of azoxymethane (AOM, Sigma, A5486) per gram body weight was injected intraperitoneally in control and constitutive *Pygo2* knockout mice. Seven days after AOM treatment, DSS 1.5% (w/v) was supplied in drinking water for five continuous days. Mice were sacrificed six months after the onset of AOM treatment. The intestine was cut open longitudinally and tumor numbers and sizes were recorded.

### Epithelial cell extraction from small intestine and colon

Isolation of intestinal epithelial cells from mice was performed as previously described [94, 95] with some modifications. Immediately after sacrificing, the intestines were cut longitudinally and rinsed in PBS. Further, they were incubated in a HBSS/EDTA (pH 7.4) solution at 37°C for 15 minutes to destabilize the outer epithelial layer of the intestine. To extract the epithelial cells into the HBSS solution, tissues were vortexed for 2 min and cells were collected by centrifugation at 1600 rpm for 10 min. Finally, the pellet containing predominantly intestinal epithelial cells were stored in 1ml of TRIZOL at −80°C until use.

### Immunohistochemistry (IHC)

All tissues were fixed in 4% PFA and 3-micron tissue sections were used. In all experiments, standardized protocols were used. For BrdU for *in vivo* DNA labeling, mice were injected intraperitoneally 2 hours prior to sacrificing with 100μg BrdU (Roche, 10280879001) per gram body weight. For immunohistochemistry, antigen retrieval of tissue sections was performed by heating in 10 mM Tris, 1 mM EDTA (pH 9.0). After blocking, sections were incubated overnight with respective primary antibodies (see Supplementary Table S2). HRP conjugated secondary antibodies were applied and the staining visualized by DAB according to manufacturer's protocol.

### cDNA synthesis and qRT-PCR

RNA was isolated using Tri-Reagent (Ambion-Invitrogen) and reverse-transcribed using MMLV-RT (Thermo Fisher Scientific). qRT-PCR was performed with SYBR green (Sigma-Aldrich) on the ABI Prism 7900HT (Applied Bio-systems). Primers used in the study are listed in Supplementary Table S1. Relative gene expression was calculated using HPRT as a housekeeper gene.

### Microscopy

All bright field images were acquired by an OLYMPUS SC30 integrated to an OLYMPUS bx43 microscope. CellSens Dimension 1.6 software was utilized for image acquisition.

### Statistical analysis

Data of qRT-PCRs were analyzed using Microsoft excel. For statistical significance, the two-sided student *t*-test was used. Graph Pad Prism6 assisted Kaplan-Meier survival curve was used to compare the survival time of mice. Box plot analysis was performed for the comparison of tumor size and number of chemically induced tumors.

## SUPPLEMENTARY MATERIALS


