# Supplementary material for: The role of Pygo2 for Wnt/ß-catenin signaling activity during intestinal tumor initiation and progression
Source: Oncotarget. 2016 Nov 2;7(49):80612–32. doi: 10.18632/oncotarget.13016 (PMC5348345; doi:10.18632/oncotarget.13016)
Supplement: Supplementary file 1 [file oncotarget-07-80612-s001.pdf]

The role of *Pygo2* for Wnt/ß-catenin signaling activity during intestinal tumor initiation and progression

Supplementary Materials

A

genotype	body weight [g]					weight change [%]	
Pygo2 wildtype	start of treatment d0	end of treatment d7	recovery phase d14	recovery phase d21	recovery phase d28	d0 to d7	d0 to d28
# 1	31,0	26,0	27,0	28,0	31,4	-16,1	1,3
# 2	33,4	29,0	32,2	33,0	33,0	-13,2	-1,2
# 3	28,0	23,0	25,6	25,0	26,0	-17,9	-7,1
# 4	32,0	26,0	30,3	31,0	33,0	-18,8	3,1
# 5	25,0	20,0	23,0	23,4	23,8	-20,0	-4,8
**mean**	**31,0**	**26,0**	**27,0**	**28,0**	**31,4**	**-17,9**	**-1,2**
± *SEM*	*3,0*	*3,1*	*3,3*	*3,6*	*3,8*	*2,4*	*3,8*
Pygo2 knockout	start of treatment d0	end of treatment d7	recovery phase d14	recovery phase d21	recovery phase d28	d0 to d7	d0 to d28
# 1	29,6	25,5	27,0	27,8	30,0	-13,9	1,4
# 2	25,6	21,0	25,0	26,0	26,0	-18,0	1,6
# 3	28,0	23,0	27,0	28,0	29,7	-17,9	6,1
# 4	31,0	25,0	28,0	29,0	30,0	-19,4	-3,2
# 5	26,0	21,0	23,0	23,0	25,0	-19,2	-3,8
# 6	24,7	21,0	22,0	23,0	25,0	-15,0	1,2
**mean**	**27,0**	**22,0**	**26,0**	**26,9**	**27,9**	**-17,9**	**1,3**
± *SEM*	*2,3*	*1,9*	*2,2*	*2,4*	*2,3*	*2,1*	*3,3*

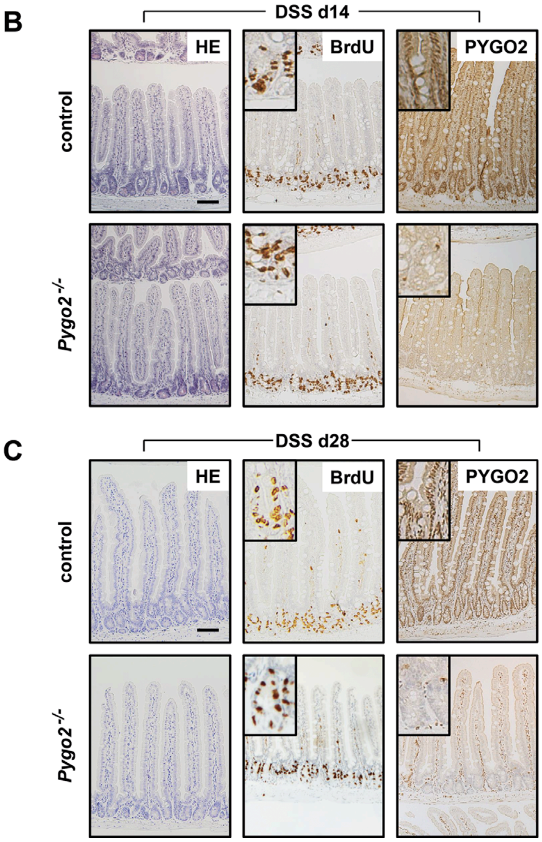

Supplementary Figure S1: *Pygo2* is dispensable for intestinal regeneration following chemically induced inflammation. (**A**) Representative evaluation of the body weight development of a matched group of litter mates mice (control and Pygo2 knockout) following treatment with dextran sodium sulphate for five consecutive days (1.5% w/v DSS). (**B**, **C**) Representatives examples of immunohistochemical stains on intestinal tissues of controls (labelled with control, i.e. $Pygo2^{lox(ex3)/lox(ex3)}$) and constitutive *Pygo2* deficient littermates ($Pygo2^{-/-}$; corresponding to Vil^{Cre}; $Pygo2^{\Delta/\Delta}$). Animals were analyzed after 14 (B) and 28 days (C) from the beginning of DSS treatment. In mutant animals, PYGO2 expression was not detectable throughout the entire intestinal epithelium. Despite the constitutive knockout of *Pygo2*, the intestinal epithelium was completely restored as in control animals at both points of time as indicated by H&E stains and BrdU analysis.

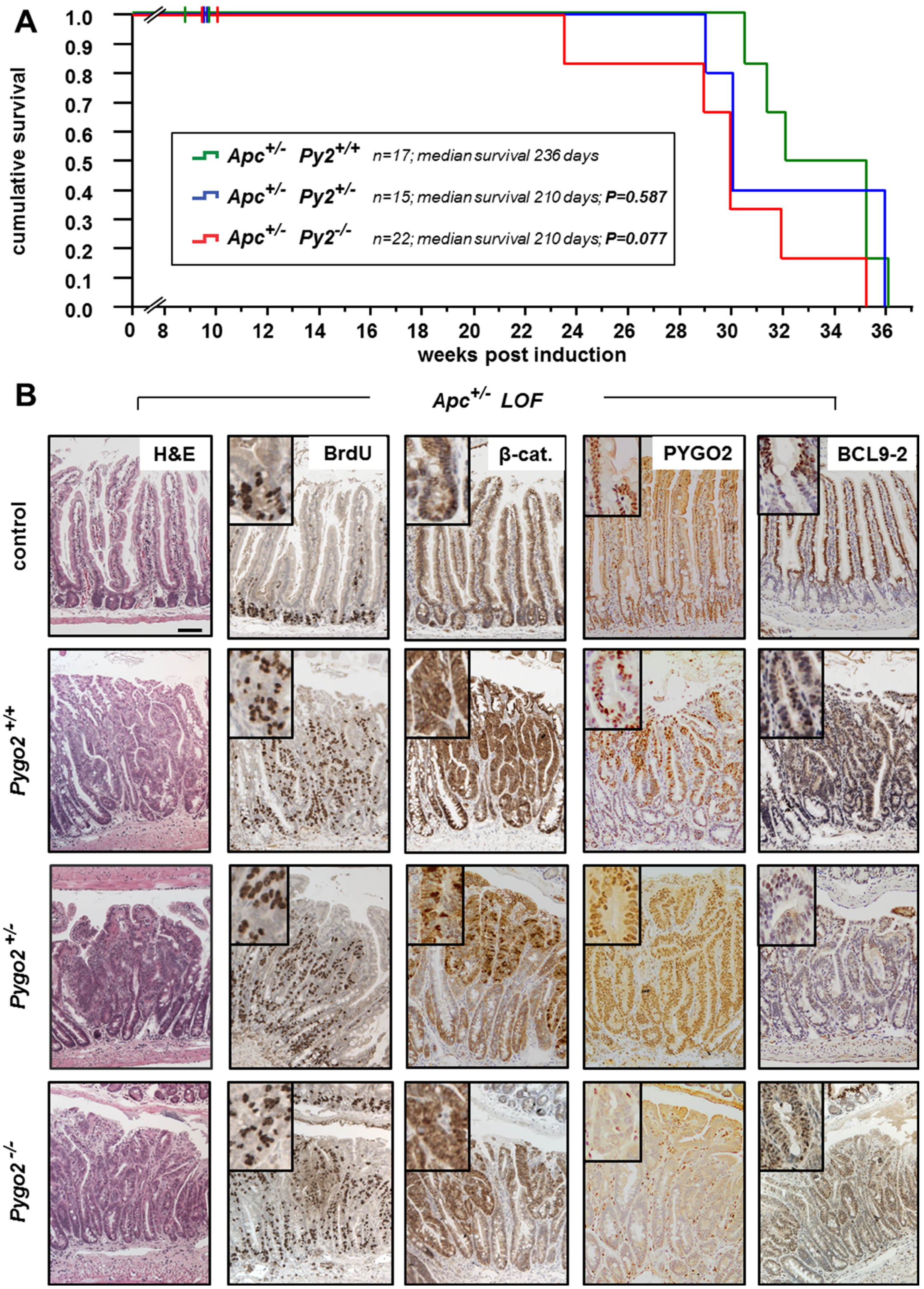

Supplementary Figure S2: Knockout of *Pygo2* does not rescue adenoma formation induced by heterozygous *Apc* loss-of-function. (**A**) Kaplan-Meier survival analysis of compound heterozygous *Apc* loss-of-function animals (*LOF*) with wild type *Pygo2* expression (labelled with APC$^{+/-}$Py2$^{+/+}$ corresponding to the genotype *Vil*$^{Cre\text{-}ERT}$*; Apc*$^{\Delta ex15/+}$*; Pygo2*$^{+/+}$*, n* = 17) compared to heterozygous *Pygo2* deficient mice (marked with APC$^{+/-}$Py2$^{+/-}$ corresponding to *Vil*$^{Cre\text{-}ERT}$*; Apc*$^{\Delta ex15/+}$*; Pygo2*$^{\Delta/+}$*; n* = 15) and homozygous *Pygo2* knockout mice (labelled with APC$^{+/-}$; Py2$^{-/-}$ corresponding to *Vil*$^{Cre\text{-}ERT}$*; Apc*$^{\Delta ex15/+}$*; Pygo2*$^{\Delta/\Delta}$; *n* = 22). All mice were induced by tamoxifen injection for five consecutive days, and survival was monitored for the indicated time. Statistics were calculated by P Log Rank Test. (**B**) Immunostains on intestinal sections from compound heterozygous *Apc LOF* mutant animals with wild type *Pygo2* expression (*Pygo2* $^{+/+}$) compared to mice with hetero- and homozygous ablation of *Pygo2* (*Pygo2* $^{+/-}$ and *Pygo2* $^{-/-}$). Animals were analyzed on day seventy after start with tamoxifen treatment. The upper panel shows intestinal tissues from control animals (*Apc*$^{lox(ex15)/+}$*; Pygo2*$^{lox(ex3)/lox(ex3)}$). Tissue sections were stained by H&E and with the indicated antibodies. Scale bars in the pictures represent 200 µm for all IHC. Inserts show the staining at higher magnification.

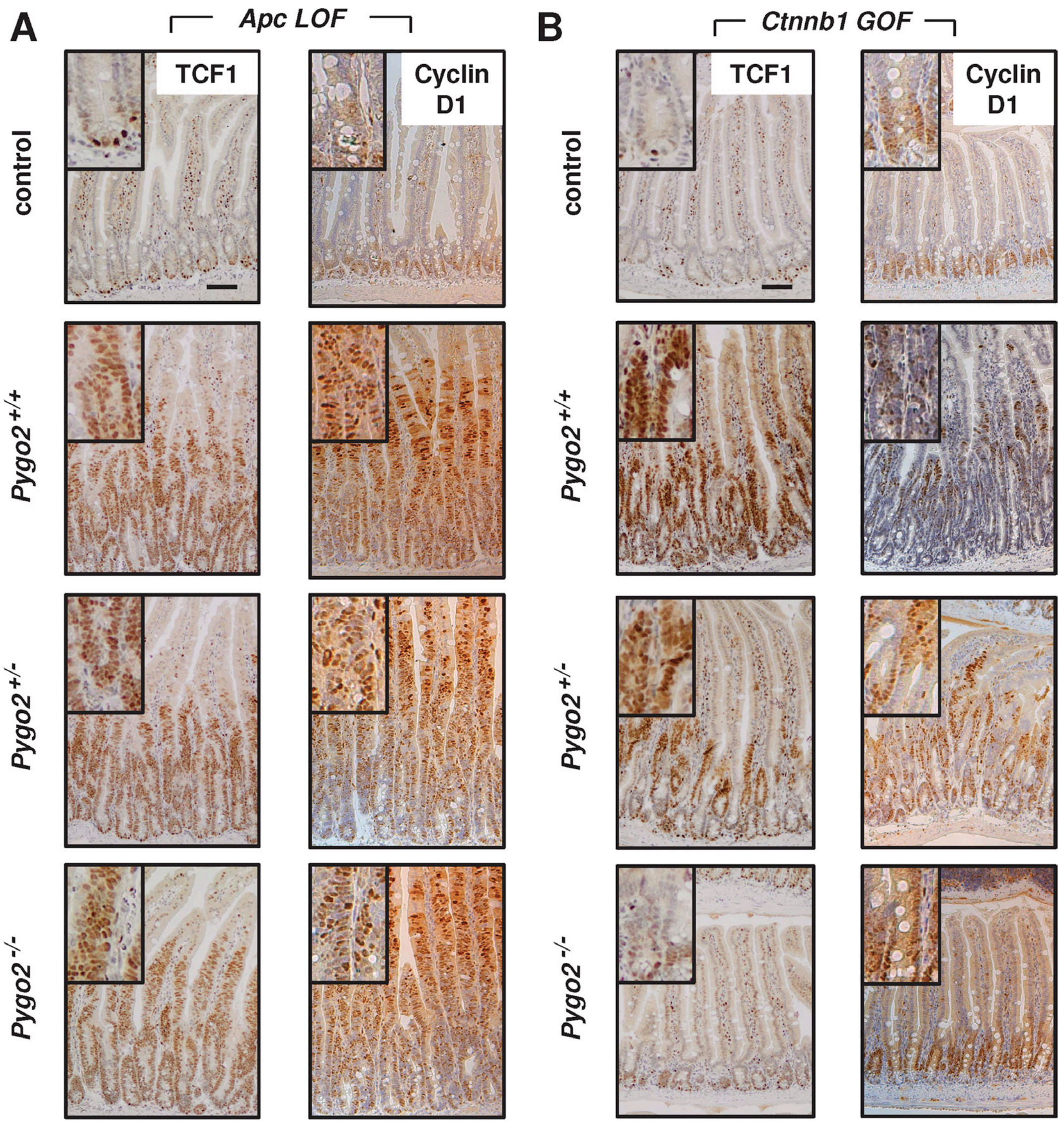
A
Apc LOF
B
Ctnnb1 GOF
TCF1
Cyclin D1
TCF1
Cyclin D1
control
Pygo2+/+
Pygo2+/-
Pygo2-/-
control
Pygo2+/+
Pygo2+/-
Pygo2-/-

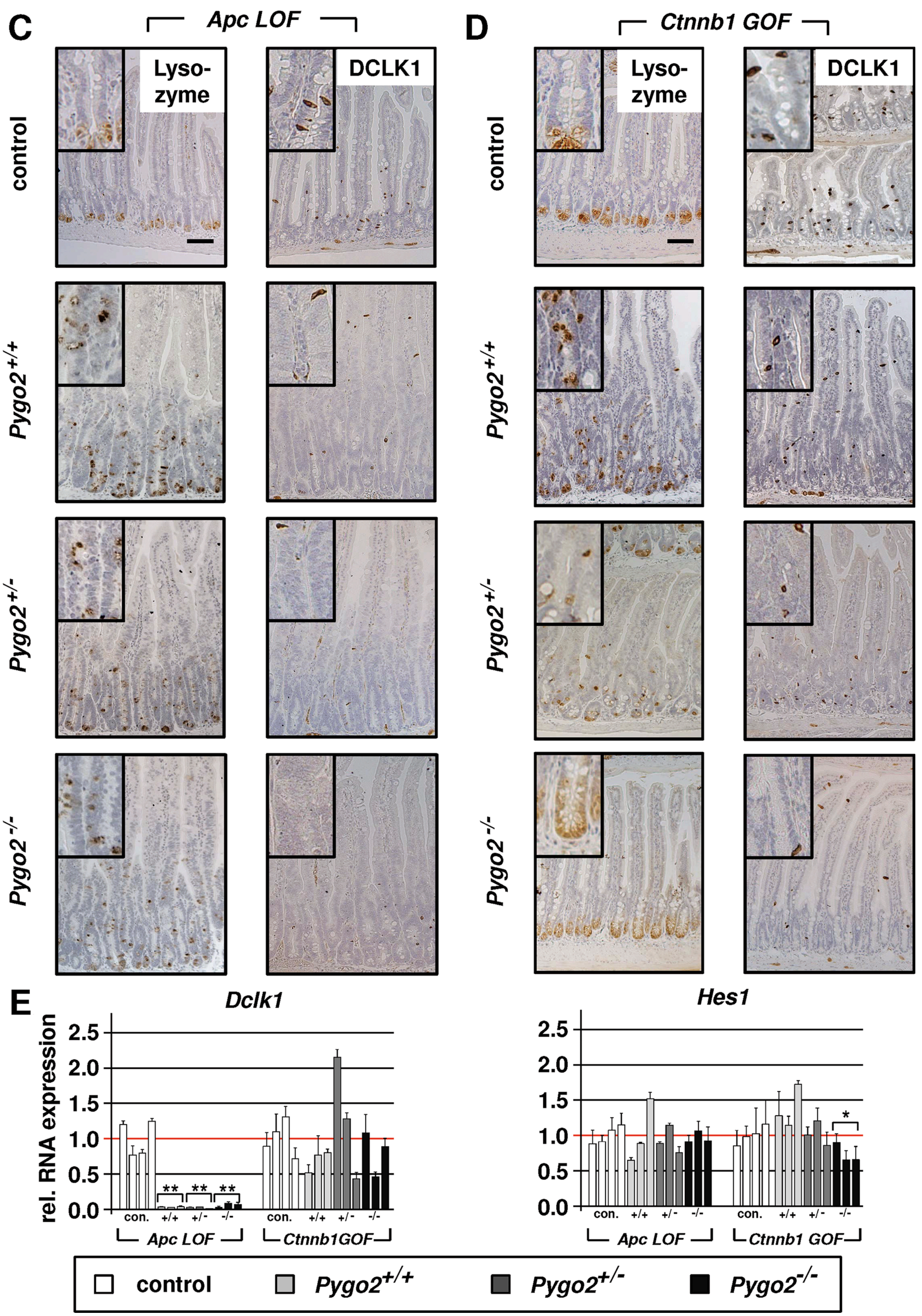

Supplementary Figure S3: The effect of *Pygo2* ablation on Wnt/ß-catenin target genes, differentiation marker expression and on Notch signaling during intestinal hyperproliferation induced by *Apc LOF* and *Ctnnb1 GOF*. (**A**, **B**) Representative pictures of immunostains for the Wnt/ß-catenin target genes TCF1 and CyclinD1 and (**C**, **D**) for the differentiation markers including Lysozyme (Paneth cells) and DCLK1 (Tuft cells) detection. Intestinal tissue sections were analyzed of mice with the indicated genotypes from compound *Apc LOF* (A, C) and *Ctnnb1 GOF* animals (B, D). (**E**) qRT-PCR analyses for the relative RNA expression of *Dclk1* and *Hes1*, a Notch target gene. Each graph represents the mean of at least three independent experiments from RNA of mice with the indicated genotype. Significant differences for the mean expression level relative to the corresponding control group is marked * for $P < 0.05$, and ** for $P < 0.01$.

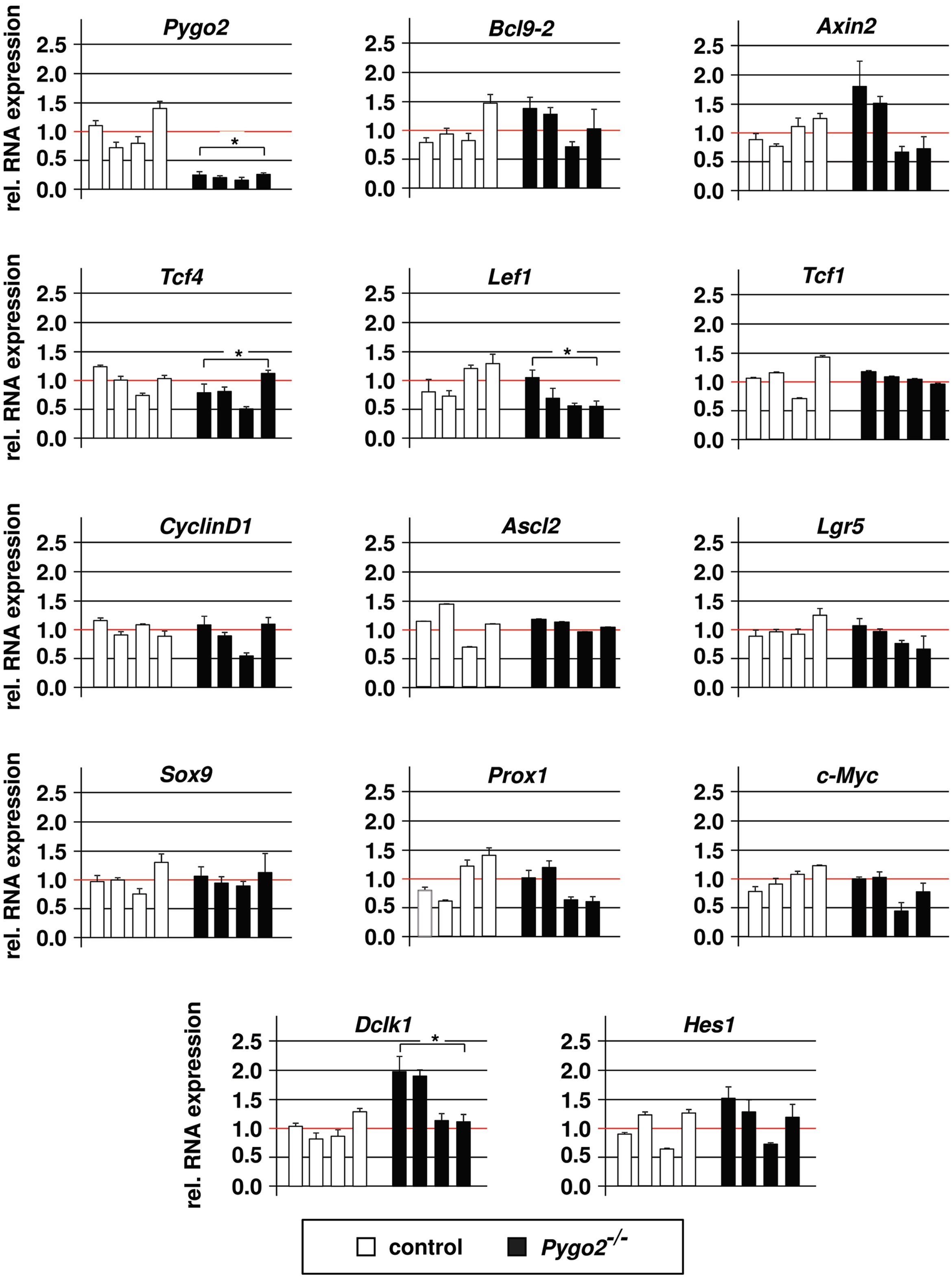

Supplementary Figure S4: The effect of *Pygo2* knockout on target gene expression in the normal intestine. qRT-PCR analyses of RNA extracted from epithelial cells of the intestine of controls (control: *Pygo2*$^{lox(ex3)/lox(ex3)}$) compared to homozygous *Pygo2* deficient animals (*Pygo2*$^{-/-}$: *Vil*$^{Cre\text{-}ERT}$; *Pygo2*$^{\Delta/\Delta}$). Mice were analyzed on day 18 post induction with tamoxifen. The graphs show the relative RNA expression of the indicated gene; each bar represents one animal. Significances were calculated for the mean expression level compared to the control group. The asterisk marks significant differences * for $P < 0.05$.

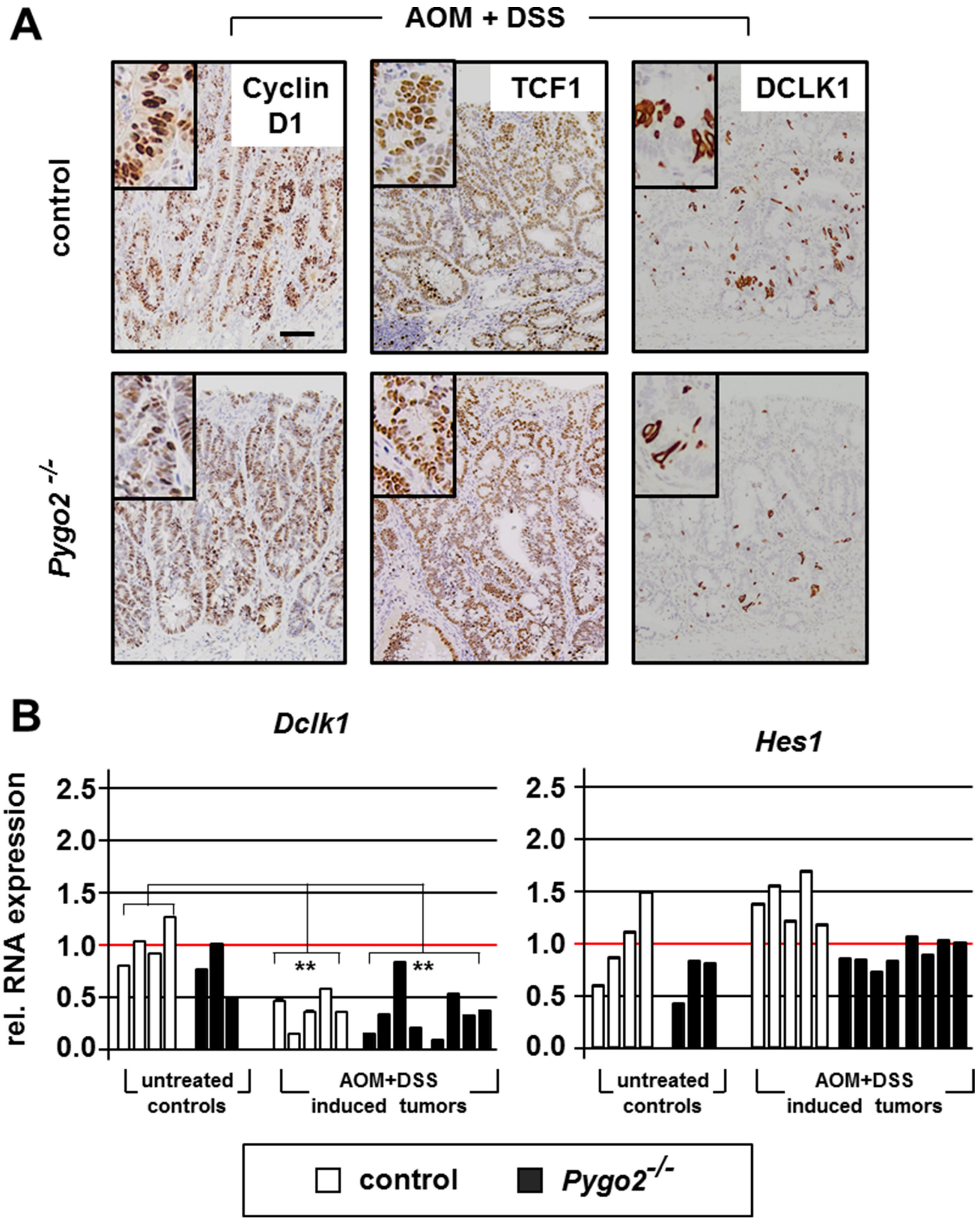

Supplementary Figure S5: The effect of *Pygo2* knockout on Wnt/ß-catenin target genes, differentiation marker expression and on Notch signaling in chemically induced intestinal tumors. (**A**) Immunostains for the detection of Wnt/ß-catenin target genes TCF1, CyclinD1 and the differentiation marker DCLK1. Tumor sections were from controls and *Pygo2* knockout animals. Mice were sacrificed six months after treatment with AOM and DSS. (**B**) qRT-PCR analyses for the relative RNA expression of *Dclk1* and *Hes1* in tumors and from colon tissues of untreated mice, both of controls and *Pygo2* deficient animals. Each graph shows the relative RNA expression of the indicated gene. Significances were calculated for the mean expression level compared to untreated controls and relative to the expression levels of control tumors, respectively. ** marks significant differences between the respective groups with $P < 0.01$.

Supplementary Table S1: qRT-PCR primer

Target gene primer name	Sequence (5′–3′)
Pygo2-S	GGTTGAGCAGAGCCATTCCT
Pygo2-AS	CAGCCATGGGGCTATACAGG
Bcl9-2-S	AATCATGGCAAGACAGGGAATGGA
Bcl9-2-AS	TCTTCAGACTTGAGTTGCTAGGTG
Axin2-S	GCTCCAGAAGATCACAAAGAGC
Axin2-AS	AGCTTTGAGCCTTCAGCATC
c-Myc-S	GACCTAACTCGAGGAGGAGCTGGAATC
c-Myc-AS	AAGTTTGAGGCAGTTAAAATTATGGCTGAAGC
Sox9-S	ACTCCCCACATTCCTCCTCC
Sox9-AS	GGACCCTGAGATTGCCCAGA
Prox1-S	GCTCCAACATGCTGAAGACCTA
Prox1-AS	GCTGCGAGGTAATGCATCTG
Lgr5-S	CCAATGGAATAAAGACGACGGCAACA
Lgr5-AS	GGGCCTTCAGGTCTTCCTCAAAGTCA
Lef1-S	AGAACACCCTGATGAAGGAAAG
Lef1-AS	GTACGGGTCGCTGTTCATATT
Tcf4-S	AAGACTTGAACATTAGCGAGAG
Tcf4-AS	AAGAGCACAGGGCAGTTG
Dcmkl1-S	CAGCAAGTCTCCCAGAAGATAC
Dcmkl1-AS	AGGACTGGAGACCACACTAA
Ccnd1 (CyclinD1)-S	TGCGTGCAGAAGGAGATTGT
Ccnd1 (CyclinD1)-AS	CCTCACAGACCTCCAGCATC
Hes1-S	CATGGAGAAGAGGCGAAGGG
Hes1-AS	GGAATGCCGGGAGCTATCTT
Ascl2-S	AAG CAC ACC TTG ACT GGT ACG
Ascl2-AS	AAG TGG ACG TTT GCA CCT TCA
HPRT-S	CCTAAGATGAGCGCAAGTTGAA
HPRT-AS	CCACAGGACTAGAACACCTGCTAA
Tcf1-S	TCTGCTCATGCCCTACCCA
Tcf1-AS	TGTTATGCAGCGGGGGTTGAG

All oligonucleotides were synthesized by IBA GmbH, Göttingen, and Metabion AG, München.

Supplementary Table S2: Antibodies for immunohistochemical analyses

Antibody	Source	Dilution	Catalogue No.	Manufacturer
Anti-ß-catenin	Mouse	1:500	610154	BD Transduction
Anti-ß-catenin	Rabbit	1:2000		[3]
Anti-BrdU	Rat	1:100	ab6326	Abcam
Anti-Pygo2	Rabbit	1:1500		[3]
Anti-BCL9-2	Rabbit	1:250		[3]
Anti-BCL9	Rabbit	1:1500		[3]
Anti-DCLK1	Rabbit	1:1000	ab31704	Abcam
Anti-Sox9	Rabbit	1:3000	AB5535	Millipore
Anti-Prox1	Rabbit	1:300	102-PA32AG	ReliaTech GmbH
		1:500	ab11941	Abcam
Anti-c myc	Rabbit	1:400	06-340	EMD Millipore
Anti-Lysozyme	Rabbit	1:2000	18-0039	Invitrogen
Anti-Tcf4	Rabbit	1:500	2565S	Cell signaling
Anti-CyclinD1	Rabbit	1:300	2978S	Cell signaling
Anti-Lef1	Rabbit	1:500	C12A5	Cell signaling
Tcf1	Rabbit	1:400	C63D9	Cell signaling
Anti-Lgr5	Rabbit	1:500	AP03046U-N	Acris antibodies
